# Recombinant Klotho administration after myocardial infarction reduces ischaemic injury and arrhythmias by blocking intracellular calcium mishandling and CaMKII activation

**DOI:** 10.1002/path.6388

**Published:** 2025-01-15

**Authors:** Sara Vázquez‐Sánchez, Ana Blasco, Pablo Fernández‐Corredoira, Paula Cantolla, Elisa Mercado‐García, Elena Rodríguez‐Sánchez, Laura González‐Lafuente, Jonay Poveda, Daniel González‐Moreno, Andrea Matutano, Sonia Peribañez, Inés García‐Consuegra, Massimo Volpe, María Fernández‐Velasco, Luis M. Ruilope, Gema Ruiz‐Hurtado

**Affiliations:** ^1^ Cardiorenal Translational Laboratory Imas12 Research Institute, Hospital Universitario 12 de Octubre Madrid Spain; ^2^ RICORS2040‐Renal Madrid Spain; ^3^ Acute Cardiac Care Units Cardiology Service. Hospital Universitario Puerta de Hierro‐Majadahonda Madrid Spain; ^4^ Research Ethics Committee Instituto de Investigación Puerta de Hierro‐Segovia de Arana Madrid Spain; ^5^ Cardiology Service Hospital Universitario Miguel Servet Zaragoza Spain; ^6^ Clinical and Invasive Cardiology Group, Instituto de Investigación Sanitaria del Hospital La Paz (IdiPAZ) Hospital Universitario La Paz Madrid Spain; ^7^ Proteomics Unit Institute of Research Imas12, Hospital Universitario 12 de Octubre Madrid Spain; ^8^ Department of Clinical and Molecular Medicine Sapienza University of Rome Rome Italy; ^9^ IRCCS San Raffaele Rome Italy; ^10^ Centro de Investigación Biomédica en Red de Enfermedades Cardiovasculares (CIBER‐CV) Instituto de Salud Carlos III Madrid Spain; ^11^ European University of Madrid Madrid Spain; ^12^ Department of Physiology, School of Medicine Universidad Autónoma de Madrid Madrid Spain

**Keywords:** ischaemic heart disease, Klotho, arrhythmia, cardiomyocyte, CaMKII, myocardial infarction, calcium mishandling, STEMI, ryanodine

## Abstract

Ischaemic heart disease (IHD) remains a major cause of death and morbidity. Klotho is a well‐known anti‐ageing factor with relevant cardioprotective actions, at least when renal dysfunction is present, but its actions are much less known when renal function is preserved. This study investigated Klotho as a biomarker and potential novel treatment of IHD‐associated complications after myocardial infarction (MI) under preserved renal function. Association between circulating Klotho levels and cardiac injury was investigated in patients after ST‐elevation MI (STEMI). Biochemical, *in vivo* and *in vitro* cardiac function and histological and molecular studies were performed to determine the effect of recombinant Klotho in the failing hearts of mice after MI. We demonstrated that STEMI patients showed lower systemic Klotho levels, with the lowest Klotho tertile in those patients with higher N‐terminal pro B‐type natriuretic peptide (NT‐proBNP) levels. Mice also showed a decrease in systemic Klotho levels after MI induction. Furthermore, recombinant Klotho administration in mice reduced infarct area and attenuated cardiac hypertrophy and fibrosis. We also demonstrated that Klotho treatment prevented reduction in ejection fraction and MI‐related ECG changes, including prolonged QRS, JT, QTc, and T_peak_T_end_ intervals and premature ventricular contractions. In adult mouse cardiomyocytes, Klotho treatment restricted systolic calcium (Ca^2+^) release and cell shortening disturbances after MI. Klotho prevented increased diastolic Ca^2+^ leak and pro‐arrhythmogenic events in PMI mice by blocking activation of the Ca^2+^/calmodulin‐dependent kinase type II (CaMKII) pathway, preventing ryanodine receptor type 2 (RyR_2_) hyperphosphorylation. In conclusion, Klotho supplementation protected against functional and structural cardiac remodelling and ameliorated ventricular arrhythmic events by preventing intracardiomyocyte Ca^2+^ mishandling in mice following MI. These data uncover a new cardioprotective role of Klotho, emerging as a biomarker of ventricular injury and potential treatment for patients after MI. © 2025 The Author(s). *The Journal of Pathology* published by John Wiley & Sons Ltd on behalf of The Pathological Society of Great Britain and Ireland.

## Introduction

Cardiovascular disease is the leading cause of death and disability worldwide, and ischaemic heart disease (IHD), caused by coronary artery disease or myocardial infarction (MI), remains the leading cause of cardiovascular mortality despite enormous efforts to improve survival rates in recent years [1]. Clinical advances have significantly reduced mortality after MI; however, long‐term IHD can lead to the development and progression of heart failure (HF), which is a major cause of chronic cardiovascular morbidity and mortality [2]. The development of HF after MI is caused by several mechanisms, including necrosis and scar formation, cardiomyocyte injury and death, neurohumoral activation, and detrimental remodelling of the viable ventricle to compensate for the remaining dead tissue [3]. Ventricular remodelling is characterised by cardiomyocyte hypertrophy and interstitial fibrosis, which alter the electrical coupling between cells, increasing the likelihood of ventricular arrhythmias and sudden cardiac death [4, 5]. After MI, patients often experience an acute phase of ventricular tachycardia and arrhythmic events, which is a common clinical complication associated with high in‐hospital mortality [6]. At the cellular level, post‐MI (PMI) HF can lead to impaired intracardiomyocyte calcium (Ca^2+^) handling. This can cause abnormal systolic and diastolic Ca^2+^ release, leading to contractile dysfunction and an increased risk of cellular arrhythmias [7, 8, 9].

The search for new therapeutic strategies for the early prevention of HF and arrhythmic complications after MI is ongoing. Although several available therapeutic interventions have been shown to provide significant clinical benefit after MI and are widely used in clinical practice (renin‐angiotensin‐aldosterone system inhibitors, beta blockers, statins, antiplatelet agents, and non‐pharmacological therapy), ischaemic HF remains the leading cause of death in developed countries, indicating the need for more effective therapies. Klotho is a recently discovered protein that suppresses several ageing phenotypes [10] and has pleiotropic effects on multiple cardiometabolic processes [11, 12], representing a promising therapeutic option for managing ischaemic HF. A transmembrane form of Klotho is expressed in several organs and tissues, with the highest levels found in the kidney and much lower levels in the lung, liver, skeletal muscle, aorta, and brain [10, 11]. Klotho can be cleaved by anchored proteases in the extracellular medium and released into the systemic circulation, giving rise to its soluble form, which also acts in a paracrine manner in various target tissues [13, 14, 15]. Soluble Klotho has been reported to exert several systemic beneficial effects, including anti‐inflammatory, antioxidant, antifibrotic, and anti‐apoptotic effects in several metabolic disorders [16, 17]. Moreover, recent studies have demonstrated that increasing the bioavailability of circulating Klotho levels by recombinant Klotho supplementation or genetic Klotho overexpression can have cardioprotective effects by preventing cardiac dysfunction in several experimental models of cardiorenal syndrome [18, 19, 20]. In humans, low levels of circulating Klotho are associated with the presence and severity of coronary artery disease independent of classical cardiovascular risk factors [21]. However, the effects and mechanisms of Klotho as a potential therapeutic option in the context of ischaemic HF remain controversial and poorly understood.

Here, we investigated the relationship between circulating Klotho levels and cardiac injury in patients after ST‐elevation myocardial infarction (STEMI) and in an experimental model of PMI. In addition, we tested the effect of exogenous Klotho by recombinant murine Klotho treatment in PMI mice and analysed its overall cardiac benefits on cardiac function, fibrosis, cardiomyocyte contraction, Ca^2+^ handling, and ventricular rhythmic events. We also investigated the intracellular mechanisms involved in these effects.

## Materials and methods

### Ethical approval for the human study

The human study was approved by the Human Ethics Committee of the Hospital Universitario 12 de Octubre (Madrid, Spain; reference: 16/250) and was performed in accordance with the Declaration of Helsinki. Written informed consent was obtained from all patients. For the cohort of patients from the Hospital Universitario Miguel Servet (Zaragoza, Spain), samples and data were provided by the Biobank of the Aragon Health System (PT20/00112), integrated in the Spanish National Biobanks Network. Patient samples and data were also provided by the Hospital Universitario Puerta de Hierro Majadahonda (HUPHM)/Instituto de Investigación Sanitaria Puerta de Hierro‐Segovia de Arana (IDIPHISA) Biobank (Carlos III Health Institute Biobanks and Biomodels Platform). All patients' samples and data were processed following standard operating procedures with appropriate approval of the Ethics and Scientific Committees.

### Systemic human Klotho levels

Plasma alpha Klotho (α‐Klotho) levels were analysed in two independent cohorts of STEMI patients with acute coronary syndrome after MI: *n* = 41 patients were recruited from the Cardiology Service of the Hospital Universitario Miguel Servet (Zaragoza, Spain), and *n* = 73 patients were recruited from the Cardiology Service of the Hospital Universitario Puerta De Hierro (Madrid, Spain). Blood samples were drawn within the first 24 h of hospital admission. Plasma α‐Klotho levels were also measured in a control group of *n* = 43 healthy blood donor volunteers without cardiovascular disease. The average age of the healthy subjects was 40.18 ± 12.61 years, with 41% male and 59% female. Plasma α‐Klotho was determined using a commercial enzyme‐linked immunosorbent assay (ELISA) kit (IBL‐America, Minneapolis, MN, USA).

### Ethical approval for animal studies

Animal studies were performed after approval was given by the Bioethical Committee of the Universidad Autónoma de Madrid and was approved by the General Direction of Agriculture and the Environment at the Environment Council of Madrid (PROEX 186.5/20) according to the Guide for the Care and Use of Laboratory Animals and Guidelines for Ethical Care and Welfare (2013/175) of Experimental Animals of the European Union (2010/63/EU).

### PMI experimental model

Adult C57BL/6J mice (12–14 weeks old) from Charles River Laboratories International (Wilmington, MA, USA) underwent experimental MI by permanent ligation of the left anterior descending (LAD) coronary artery. Sham mice were used as the control group and underwent the same procedure but without coronary ligation. Sham and PMI mice were randomly sex‐matched and assigned to two groups. Each group received daily i.p. injections of either a vehicle solution (0.9% sodium chloride) or recombinant murine Klotho (10 μg/kg/day) dissolved in the same vehicle. Recombinant α‐Klotho was sourced from R&D Systems (1819‐KL‐050; Minnesota, MN, USA). Animals were distributed into the following four experimental groups: sham‐operated mice treated with vehicle (Sham) or recombinant Klotho (Sham + KL) and PMI mice treated with vehicle (PMI) or recombinant Klotho (PMI + KL).

Cardiac magnetic resonance imaging (CMRI) was performed using a 1 T benchtop MRI scanner (ICON 1 T‐MRI; Bruker BioSpin GmbH., Ettlingen, Germany) operating at 1 Tesla. CMRI images were analysed using ImageJ version 1.49 (Rasband, W.S., National Institutes of Health, Bethesda, MD, USA, https://imagej.net/ij/, 1997–2018) software program. ECGs were registered using a small animal physiological monitoring system (Harvard Apparatus, Holliston, MA, USA). Registries were obtained for 10 min under basal condition (before surgical procedure) and 15 days after surgical procedure. Files were analysed using LabChart 7.0 software (AD Instruments, Sydney, Australia). All animals were sacrificed 15 days after surgery. Additional details are provided in Supplementary materials and methods.

### Measurement of systemic N‐terminal pro B‐type natriuretic peptide (NT‐proBNP) levels

Plasma levels of NT‐proBNP were analysed using a commercial ELISA kit following the standard protocol (Abbexa Ltd., Cambridge, UK).

### Macroscopic and microscopic parameters of PMI mice

Heart weight to tibia length ratio (HW/TL) was calculated as an index of cardiac hypertrophy. Cardiomyocyte surface area was measured as the total cell area using images obtained using a Meta Zeiss LSM 510 confocal microscope with a ×40 water‐immersion objective and numerical aperture of 1.2.

### Histology

Hearts were harvested from anaesthetised mice, perfused with 4% KCl, fixed in 4% paraformaldehyde in PBS, and embedded in paraffin. Myocardial serial tissue sections (5 μm) were stained with haematoxylin and eosin (HE) to assess morphological changes and Masson's trichrome to determine the collagen content. Slides were reviewed by an investigator unaware of the experimental grouping using light microscopy (Olympus Bx41) and quantified using ImageJ software.

### Intracellular Ca^2+^ imaging

Isolated cardiomyocytes from the remote area of MI (border and infarcted area was removed after collagenase digestion) were preloaded with Ca^2+^‐sensitive fluorescent dye Fluo‐3 AM (5 μm; Invitrogen, Carlsbad, CA, USA) for 30 min at room temperature. Images were obtained with a MetaZeiss LSM 510 confocal microscope with a ×40 water‐immersion objective and numerical aperture of 1.2. Confocal Ca^2+^ images were analysed using home‐made routines in Interactive Data Language (IDL; Research Systems Inc., Boulder, CO, USA) and ImageJ software.

### RNA isolation and RT‐qPCR

RNA was extracted using the RNeasy Mini Kit (Qiagen, Hilden, Germany). RT‐qPCR was performed using the FastStart Essential DNA Green Master (Roche, Basel, Switzerland) on the LightCycler® 480 II instrument (Roche). Additional details are provided in Supplementary materials and methods.

### Western blotting

Frozen heart tissues from mice were pulverised and heart proteins were extracted in a lysis buffer containing 0.05 M Tris, 0.32 M sucrose, 0.5% CHAPS, 0.5 μm okadaic acid, as well as protease inhibitors 0.1 M PMSF, 12 μm leupeptin, 0.2 μm aprotinin, and 0.5 M benzamidine. Homogenates were centrifuged at 4,500 rpm for 10 min at 4°C. Proteins were separated by SDS‐PAGE electrophoresis and transferred to PVDF membranes (Bio‐Rad, Hercules, CA, USA). Further details of antibody dilutions and sources can be found in Supplementary materials and methods.

### Protein kinase A activity (PKA)

PKA activity was measured using a colorimetric assay (Thermo Fisher Scientific, Waltham, MA, USA).

### Terminal deoxynucleotidyl transferase (dUTP) Nick‐end labelling (TUNEL)

TUNEL was performed using the *in situ* cell death detection kit (Ref. 11684795910, Roche). Cardiac tissue sections were deparaffinised in xylene, followed by rehydration through a graded ethanol series and rinsing with PBS. The sections were then permeabilised with 0.1% Triton X‐100% and 0.1% sodium citrate. Subsequently, the TUNEL reaction mix was applied, and sections were incubated for 1 h at 37 °C in the dark. After PBS washing, nuclei were counterstained with DAPI. Finally, samples were analysed under a confocal fluorescence microscope with an excitation at 450–500 nm with a Meta Zeiss LSM 980 confocal microscope. Apoptosis was quantified as the number of TUNEL‐positive cells.

### Lactate dehydrogenase (LDH) assay

LDH assay was performed in plasma samples using a colorimetric kit (Ref. ab102526, Abcam, Cambridge, UK). The LDH activity was measured by assessing the rate of conversion of lactate to pyruvate, which generates NADH, quantified at 450 nm.

### Statistical analyses

Data are reported as mean ± SD. Statistical significance was evaluated by analysis of variance (ANOVA) with the Newman–Keuls multiple‐comparisons test or Student's *t‐*test, as appropriate. Normality was assessed with the Kolmogorov–Smirnov test. Multivariate linear regression analysis was employed to investigate the impact of MI and age on Klotho levels in human subjects. Statistical analysis of pro‐arrhythmogenic Ca^2+^ release was performed with contingency tables and chi‐squared of individual isolated cells with and without arrhythmic events and the percentage of cells with arrhythmic events versus total cells represented. All statistical analyses were performed using OriginPro 9.0 (OriginLab, Northampton, MA, USA), GraphPad Prism 8.0 (GraphPad Software, San Diego, CA, USA) or SPSS Statistics version 22 (IBM, Armonk, NY, USA). Differences with *p* values <0.05 were considered statistically significant.

## Results

### Patients with STEMI have decreased circulating Klotho levels, which was associated with ventricular damage

**Figure 1:**
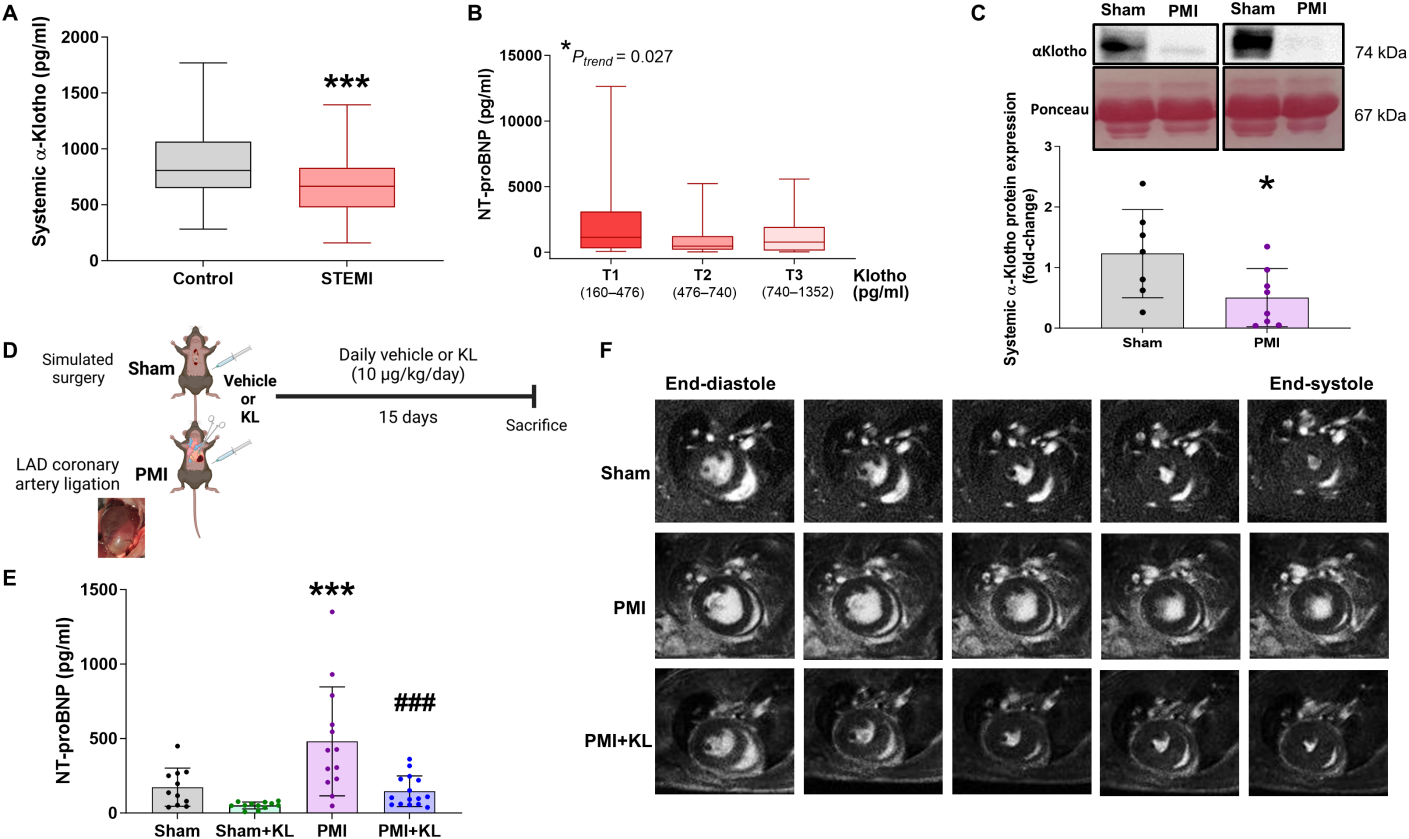
Circulating Klotho decrease after MI and their re‐establishment improves ventricular function. (A) Plasma α‐Klotho levels (pg/ml) in controls and in ST‐segment elevation myocardial infarction (STEMI) patients (*n* = 43 control, *n* = 114 STEMI patients). ***p < 0.001 versus controls. (B) NT‐proBNP levels (pg/ml) according to Klotho tertiles in STEMI patients. (C) Representative immunoblots of serum α‐Klotho levels and Ponceau S staining for total protein normalisation in sham and PMI mice 24 h after surgery, and quantification of α‐Klotho protein expression (fold‐changes) (*n* = 7 sham, *n* = 8 PMI). *p < 0.05 versus sham. (D) Experimental design (figure created with BioRender.com). (E) NT‐proBNP levels (pg/ml) in all experimental groups (*n* = 11 sham, *n* = 12 sham + KL, *n* = 13 PMI, *n* = 15 PMI + KL) (bottom panel). ***p < 0.001 versus sham. ^###^ *p* < 0.001 versus PMI. (F) Cardiac cycle MRI images (CMRI), representative two‐chamber short‐axis transverse mid‐papillary sections along the cardiac cycle from end‐diastole to end‐systole obtained in sham (upper panel), PMI (middle panel), and PMI plus Klotho treatment (bottom panel) mice. (*n* = 4 sham, *n* = 6 PMI, *n* = 6 PMI + KL). Histograms show mean values ± SD.

General demographic and cardiac data in recruited patients with STEMI are shown in Table 1. Soluble Klotho levels were significantly lower in STEMI patients than in controls (Figure 1A). Notably, STEMI patients in the lowest Klotho tertile had the highest NT‐proBNP levels (Figure 1B), with both the second and third Klotho tertiles showing significantly lower NT‐proBNP levels (Figure 1B). These results indicate that circulating Klotho levels are decreased in patients after an acute ischaemic cardiac injury, and the decrease is greater in patients with major ventricular damage. However, Klotho levels were not associated with other clinical parameters of cardiac function such as ejection fraction (EF), left ventricular end‐diastolic volume (LVEDV) or left ventricular end‐systolic volume (LVESV) in STEMI patients (data not shown). Although plasma Klotho levels may decrease with age, linear regression analysis conducted to examine the relationship between plasma Klotho levels and age revealed no significant association (*r* = −0.094; *p* = 0.143). Subsequently, an age‐adjusted model was employed to assess whether age acted as a confounding factor in the association between MI and plasma Klotho levels. Multivariate linear regression analysis indicated that, even after adjusting for age, the association between MI and plasma Klotho levels remained significant (β = −321.2; 95% CI = (−589 to −53); *p* = 0.019). Moreover, circulating Klotho levels were analysed in the presence of other relevant cardiovascular comorbidities, such as diabetes mellitus (DM), hyperlipidaemia, hypertension, or previous IHD. No statistically significant differences were found in plasma Klotho levels among STEMI patients in the presence or absence of these cardiovascular comorbidities (see supplementary material, Figure S1A–D). Furthermore, linear regression analysis revealed no association between decreased plasma Klotho levels and the presence or absence of these comorbidities (supplementary material, Table S1).

**Table 1:** Clinical data of study cohort.

Demographic characteristics	
Parameters	All patients (*n* = 114)
Age (years), mean ± SD	57.29 ± 12.20
Sex, *n* (%)	
Male	86 (75.4%)
Female	28 (24.6%)
Death, *n* (%)	10 (8.8%)
Cardiac death, *n* (%)	2 (1.8%)
Previous IHD, *n* (%)	11 (9.7%)
LVEF (%), mean ± SD	45.75 ± 10.83
BMI (kg/m^2^), mean ± SD	27.91 ± 4.5
DM, *n* (%)	18 (15.8%)
Hypertension, *n* (%)	43 (37.7%)
Dyslipidaemia, *n* (%)	55 (48.3%)
Smoking (active or former), *n* (%)	83 (72.8%)
NT‐proBNP (pg/ml), mean ± SD	1,414 ± 1941
hs‐Troponin I (ng/l), mean ± SD	64.87 ± 104.51
eGFR (ml/min/1.73 m^2^), mean ± SD	100.1 ± 10.86
Creatinine (mg/dl), mean ± SD	0.70 ± 0.16
CRP (mg/l), mean ± SD	19.67 ± 37.11

*Note*: Data are reported as mean ± SD for continuous variables and numbers (percentages) for categorical variables.

Abbreviations: BMI, body mass index; CI: confidence interval; CRP, C‐reactive protein; DM, diabetes mellitus; eGFR, estimated glomerular filtration rate; hs‐Troponin I, high‐sensitivity troponin I; IHD, ischaemic heart disease; LVEF, left ventricular ejection fraction; STEMI, ST‐elevation myocardial infarction; NT‐proBNP, N‐terminal pro‐Brain natriuretic peptide.

### Treatment with recombinant Klotho immediately after MI improves cardiac dysfunction

In the experimental IHD model, plasma Klotho levels were significantly lower in PMI mice than in sham mice 24 h after coronary artery ligation (Figure 1C). Recombinant Klotho (10 μg/kg) was administered i.p. daily for 15 days, starting immediately after MI or simulated surgery (Figure 1D). Analysis of NT‐proBNP as a marker of ventricular stress revealed that circulating levels were significantly higher in the PMI group than in the sham group, whereas levels were significantly lower in the PMI + KL group than in the PMI group (Figure 1E). We next evaluated cardiac function by CMRI. Representative short axis views of the sham, PMI, and PMI + KL groups are shown in Figure 1F, and mean values of heart rate, LVEDV, LVESV, EF, stroke volume, and cardiac output of each experimental group are shown in Table 2. We noted that EF, SV, and CO were significantly lower in the PMI group than in the sham group, whereas LVESV was significantly higher, corroborating the HF phenotype. The functional cardiac changes characteristic of the HF phenotype observed in PMI mice were significantly improved by Klotho treatment in PMI + KL mice (Figure 1F) with a significant increase in EF and SV and a significant decrease in LVESV (Table 2). These data strongly suggest that exogenous Klotho supplementation has a significant beneficial effect on preventing cardiac dysfunction and HF after MI.

**Table 2:** CMRI parameters in sham, PMI, sham + KL, or PMI + KL mice.

Parameters	Sham	PMI	Sham + KL	PMI + KL
HR (bpm)	375.9 ± 48.39	334.7 ± 45.37	383.2 ± 24.64	332.4 ± 52.48
LVEDV (μl)	41.19 ± 1.84	54.43 ± 20.18	40.13 ± 9.29	48.31 ± 11.50
LVESV (μl)	11.53 ± 2.99	32.00 ± 16.27*	7.941 ± 3.08	18.09 ± 7.85 3^#^
EF (%)	71.97 ± 7.27	48.10 ± 13.43**	79.91 ± 6.53	63.83 ± 8.22^#^
SV (μl)	29.66 ± 3.49	22.49 ± 5.35*	32.19 ± 8.07	30.22 ± 4.80^#^
CO (L/min)	11.06 ± 1.14	7.56 ± 2.17*	12.24 ± 2.80	10.18 ± 2.86

Abbreviations: Values represent mean ± SD. *n* = 4 sham, *n* = 6 PMI, *n* = 4 sham + KL, *n* = 6 PMI + KL.

Abbreviations: CO, cardiac output; EF, ejection fraction; HR, heart rate; LV, left ventricle; LVMI, LV myocardial infarction; LVEDV, left ventricular end‐diastolic volume; LVESV, left ventricular end‐systolic volume; SV, stroke volume.

* *p* < 0.05.

** *p* < 0.01 versus Sham.

^#^ *p* < 0.05 versus PMI.

### Recombinant Klotho treatment prevents alterations in systolic intracellular Ca^2+^ release and contraction in cardiomyocytes after MI

**Figure 2:**
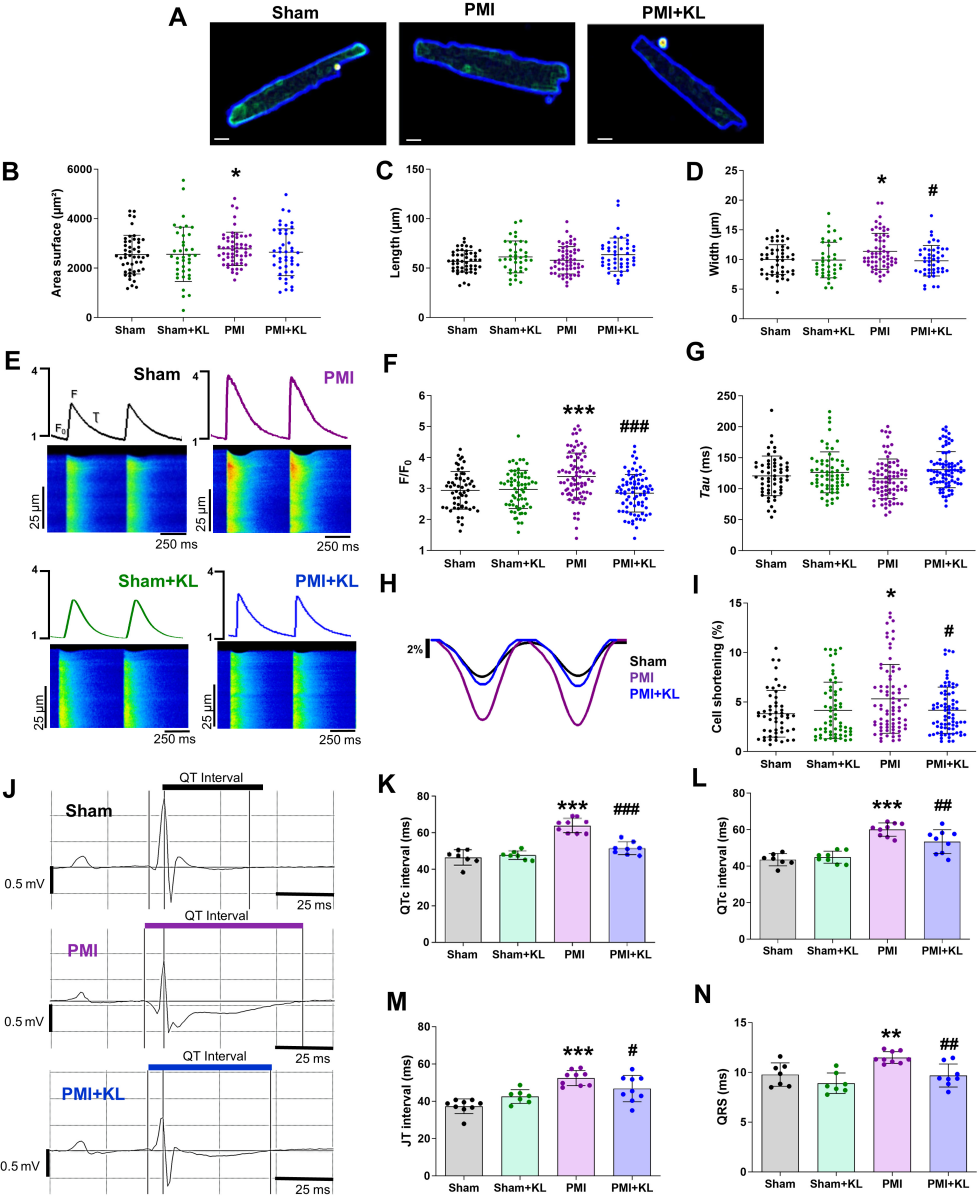
Klotho treatment attenuates cardiomyocyte hypertrophy, intracellular systolic Ca^2+^ transient mishandling, cellular contraction, and ECG changes after MI. (A) Representative images of ventricular cardiomyocytes from sham, PMI, and PMI + KL mice, scale bar = 25 μm. (B) Average cell area (C) length and (D) width values. Sham *n* = 4 mice/*n* = 49 cells; sham + KL *n* = 5 mice/*n* = 38 cells; PMI *n* = 6 mice/*n* = 57 cells; PMI + KL *n* = 6 mice/*n* = 46 cells. (E) Fluorescence profiles (upper panels) and representative images (bottom panels) of the line‐scan confocal images of systolic intracellular Ca^2+^ transients of ventricular cardiomyocytes electrically evoked by field stimulation at 2 Hz. (F) Mean values of peak fluorescence of systolic intracellular Ca^2+^ transients (amplitude F/F_0_). (G) Decay time constant (*Tau*, ms). (H) Profiles and (I) average percentages of cardiomyocyte shortening. Sham *n* = 4 mice/*n* = 52–56 cells; sham + KL *n* = 5 mice/*n* = 62–64 cells; PMI *n* = 6 mice/*n* = 75–83 cells; PMI + KL *n* = 6 mice/*n* = 75–81 cells. (J) Representative QT interval of ECG recordings in sham (upper panel), PMI (middle panel), and PMI plus Klotho (bottom panel) mice. Mean values of (K) QT, (L) QTc, (M) JT, and (N) QRS interval duration (ms) (*n* = 7–9 mice per group). Histograms represent mean ± SD. *p < 0.05, **p < 0.01, ***p < 0.001 versus sham. ^#^ *p* < 0.05, ^##^ *p* < 0.01, ^###^ *p* < 0.001 versus PMI.

Next, we characterised isolated native ventricular cardiomyocytes from the hearts of each experimental group. Representative confocal images of cardiomyocyte size from the sham, PMI, and PMI + KL groups are shown in Figure 2A. Morphometric analysis revealed that cardiomyocytes were significantly larger in the PMI group than in the sham group (Figure 2B). Cardiomyocytes from all experimental groups had a similar length (Figure 2C); however, cardiomyocytes from PMI mice were significantly wider than those from sham mice (Figure 2D). Notably, cardiomyocyte width was significantly lower in the PMI + KL group than in the PMI group (Figure 2D).

Cardiomyocyte function was analysed by examining intracellular Ca^2+^ handling. Representative line‐scan Ca^2+^ images (bottom) and their corresponding fluorescence profiles (upper panel) from cardiomyocytes of the four groups, paced at 2 Hz, are shown in Figure 2E. Systolic Ca^2+^ release was measured by analysing the fluorescence during electrical stimulation (F), basal Ca^2+^ fluorescence (F_0_), and its ratio, referred to as systolic Ca^2+^ transients' amplitude (F/F_0_). Results showed that the F/F_0_ ratio in cardiomyocytes was significantly higher in PMI mice than in sham mice (Figure 2F), indicating that the former released more Ca^2+^ in systole during electrical stimulation. Contrastingly, the F/F_0_ ratio in cardiomyocytes was significantly lower in PMI + KL mice than in PMI mice and was similar to values in sham or Sham + KL mice (Figure 2F). No significant differences were found for the time constant of Ca^2+^ transient decay (*Tau*), the time from maximum liberation Ca^2+^ during systole (F) to basal levels (F_0_) (Figure 2G), between groups, indicating that the capacity of the sarco/endoplasmic reticulum Ca^2+^‐ATPase 2a to pump back Ca^2+^ into the sarcoplasmic reticulum (SR) was similar and was unaffected 15 days after MI.

We also analysed the SR Ca^2+^ content by rapid caffeine application, which is represented in Supplementary materials and methods, supplementary material, Figure S2A. The average amplitude (F/F_0_) of caffeine‐evoked Ca^2+^ transients was not significantly different between groups (supplementary material, Figure S2B). However, caffeine‐evoked Ca^2+^ transient decay (*Tau*) was significantly slower in cardiomyocytes of PMI mice than in cardiomyocytes of sham mice (supplementary material, Figure S2C). A similar pattern was observed for fractional release in electrically evoked beats by normalising Ca^2+^ transient amplitude by the SR Ca^2+^ load in cardiomyocytes from PMI and sham mice (supplementary material, Figure S2D). These results suggest that cardiomyocytes from PMI mice likely have a higher Ca^2+^ leak from the SR, indicating the increased availability of cytosolic Ca^2+^ for cell contraction. Given this, we examined whether the changes in systolic Ca^2+^ release induced changes in cardiomyocyte contraction. Representative cell shortening profiles of cardiomyocytes from all groups at 15 days following the surgical procedure are shown in Figure 2H. Results showed that cardiomyocyte cell shortening was significantly higher in PMI mice than in sham mice (Figure 2I). This phenomenon might be a compensatory mechanism of viable cardiomyocytes to improve the function of the infarcted area and necrotic tissue. Of note, Klotho treatment prevented changes in the decay time constant (*Tau*) of caffeine Ca^2+^ transients (supplementary material, Figure S2A,C) and in the fractional release (supplementary material, Figure S2D) compared with PMI mice, with values similar to those in sham and sham + KL mice. Similarly, the increased cardiomyocyte contraction observed in cardiomyocytes from PMI mice was not evident in cardiomyocytes from PMI + KL (Figure 2I). Overall, these results provide the first evidence that recombinant Klotho administration can prevent changes in systolic Ca^2+^ release and cardiomyocyte contraction following MI.

### Recombinant Klotho treatment prevents long QT phenotype and prolonged QRS after MI

Next, we explored the potential impact of Klotho in cardiac electrical conduction. Representative ECG recordings from sham, PMI, and PMI + KL mice with the corresponding QT interval are shown in Figure 2J. The QT interval on ECG represents the duration of the entire ventricular depolarisation and repolarisation process, and the QTc interval is the QT corrected for HR. The JT interval represents repolarisation duration independent of the QRS duration being a specific repolarisation time parameter. Prolongation of QT, QTc, and JT intervals indicates defects in repolarisation, one of the main consequences of which is an increased likelihood of cardiac arrhythmias. The results showed that QT, QTc, and JT intervals were significantly longer in PMI mice than in sham mice (Figure 2K–M). The QRS complex represents ventricular depolarisation, and an increase in its duration may be indicative of cardiac hypertrophy and fibrosis. The QRS interval was significantly longer in PMI mice than in sham mice (Figure 2N), suggesting the presence of deleterious cardiac remodelling and fibrosis. Notably, Klotho treatment prevented these ECG alterations in QT, QTc, JT, and QRS intervals after MI, and all were significantly shorter in PMI + KL mice than in PMI mice (Figure 2K–N). These results indicate that Klotho can prevent the prolongation of several ECG intervals related to altered cardiac depolarisation and repolarisation after MI.

### Recombinant Klotho treatment reduces ventricular infarction area, cardiac hypertrophy, and fibrosis after MI

**Figure 3:**
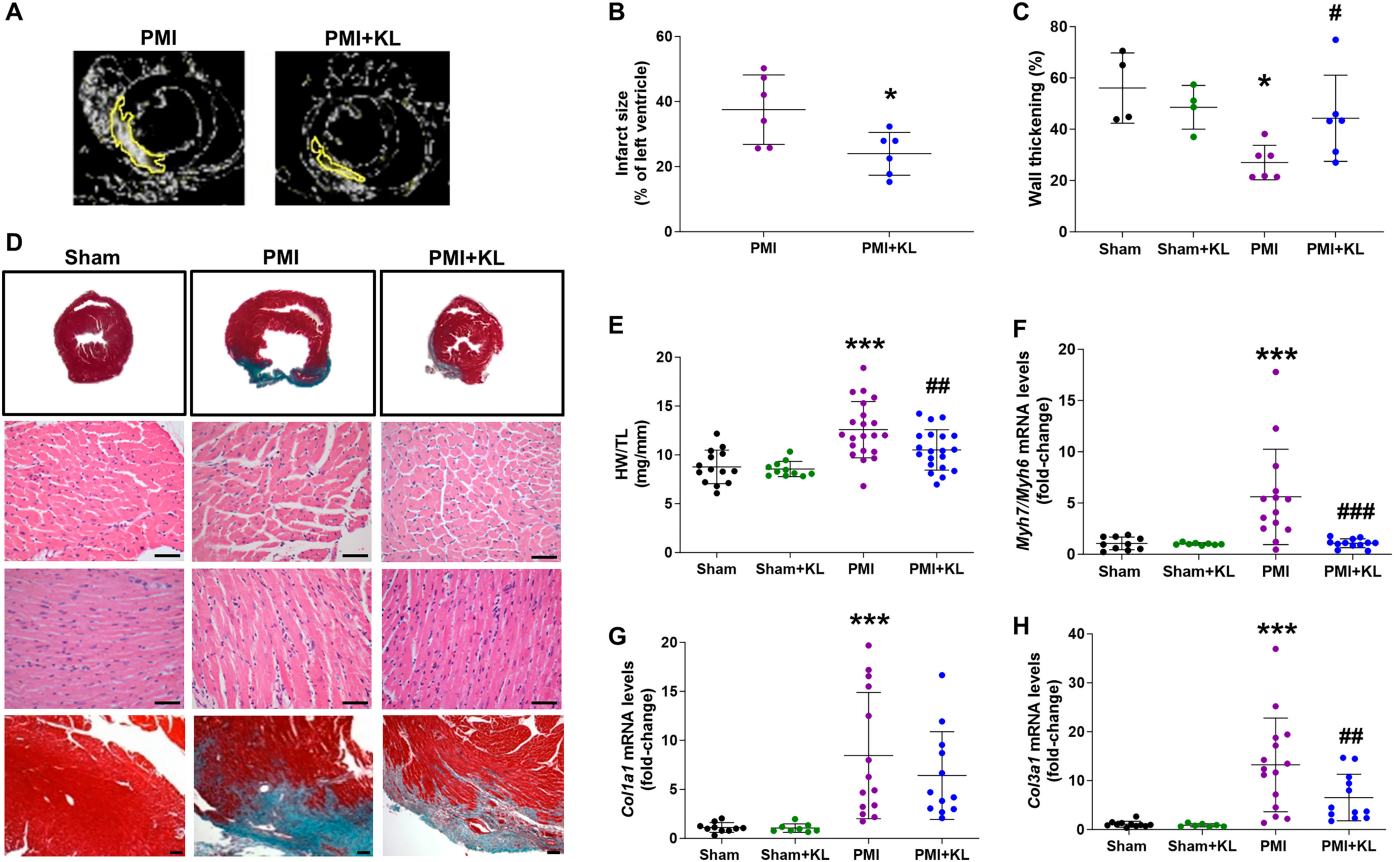
Klotho treatment limits infarct size, cardiac hypertrophy, and fibrosis in ischaemic cardiomyopathy after MI. (A) Example of late‐enhanced recordings following application of gadolinium contrast agent, obtained by CMRI in mice with PMI (left panel) and PMI plus Klotho treatment (right panel); white area marked in yellow represents ischaemic area of left ventricle that captures the contrast agent and (B) average quantified percentage values corresponding to infarction area. (C) Wall thickening percentage during heart contraction by CMRI (*n* = 4 sham, *n* = 4 sham + KL, *n* = 6 PMI, *n* = 6 PMI + KL). (D) Histological analysis of hearts from sham, PMI, and PMI + KL mice (left to right) with representative examples of histological images of whole hearts in axial views with Masson's trichrome staining showing infarction area (upper panel), and representative images of cardiac myocyte cross‐surface and long‐axis view of cardiac myocyte stained with haematoxylin and eosin at ×40 magnification, scale bar = 50 μm (middle panels), and representative Masson's trichrome‐stained images showing cardiac fibrosis of whole‐heart cross‐section at subvalvular level at ×10 magnification, scale bar = 100 μm (bottom panel); *n* = 3 sham, *n* = 3 PMI, and *n* = 3 PMI + KL. (E) Heart weight to tibia length ratio (HW/TL); *n* = 14 sham, *n* = 11 sham + KL, *n* = 21 PMI, *n* = 20 PMI + KL. (F–H) Expression of hypertrophic and fibrotic cardiac genes. Mean values of cardiac mRNA level of (F) β*/α‐mhc* (*Myh7*/*Myh6*) ratio (G) *collagen type I alpha 1 chain* (*Col1a1*), and (H) *collagen type* III alpha 1 chain (*Col3a1*); *n* = 10–11 sham, *n* = 7–8 sham + KL, *n* = 14–15 PMI, *n* = 12 PMI + KL. Histograms represent mean ± SD. *p < 0.05, ***p < 0.001 versus sham. ^#^ *p* < 0.05, ^##^ *p* < 0.01, ^###^ *p* < 0.001 versus PMI.

To test whether the alterations in ECG intervals were related to deleterious remodelling, we analysed infarct area, fibrosis, and hypertrophy in all groups of mice. Infarct area was determined by late gadolinium enhancement (LGE)‐CMRI, as shown in the representative example in Figure 3A. Results showed that the LV infarct area was significantly smaller in PMI + KL mice than in PMI mice (Figure 3B). CMRI‐estimated myocardial wall thickening was also analysed to evaluate wall motion, which revealed that wall thickness (%) was significantly lower in PMI mice than in sham mice (Figure 3C), characteristic of a HF phenotype and indicating less ventricular contraction. Contrastingly, wall thickness was significantly greater in PMI + KL mice than in PMI mice (Figure 3C). Next, we examined cardiac hypertrophy by histology (Figure 3D). We observed evident ventricular cardiac hypertrophy with a clear dilation of the LV chamber and a relevant infarcted area (upper panel) in PMI mice, along with the presence of cardiomyocyte hypertrophy at both the longitudinal and transversal levels (middle panels). These morphological data were corroborated at the macroscopic level, as shown by an increase in the heart weight/tibia length (HW/TL) ratio (Figure 3E), and at the molecular level, represented by an increase in the expression of classical cardiac hypertrophy genes measured as the ratio of foetal β/myosin heavy chain 7 or 6 (*Myh7*/*Myh6*) (Figure 3F). All of these features were essentially absent in PMI + KL mice (Figure 3E,F). Furthermore, analysis of interstitial myocardial fibrosis by Masson's trichrome staining (upper and bottom panel, Figure 3D) revealed greater fibrosis in PMI mice than in PMI + KL mice. In accord with this, the gene expression of collagen type I alpha 1 chain (*Col1a1*) (Figure 3G) and collagen type III alpha 1 chain (*Col3a1*) (Figure 3H) was significantly higher in PMI mice than in sham mice. Klotho treatment impeded the increase in the expression of both collagens, which was significant for *Col3a1* (Figure 3H). These data confirm Klotho treatment as a good therapeutic strategy to prevent the excessive cardiac remodelling that occurs after MI.

### Recombinant Klotho treatment prevents inflammatory profile, necrosis, and apoptosis after MI

**Figure 4:**
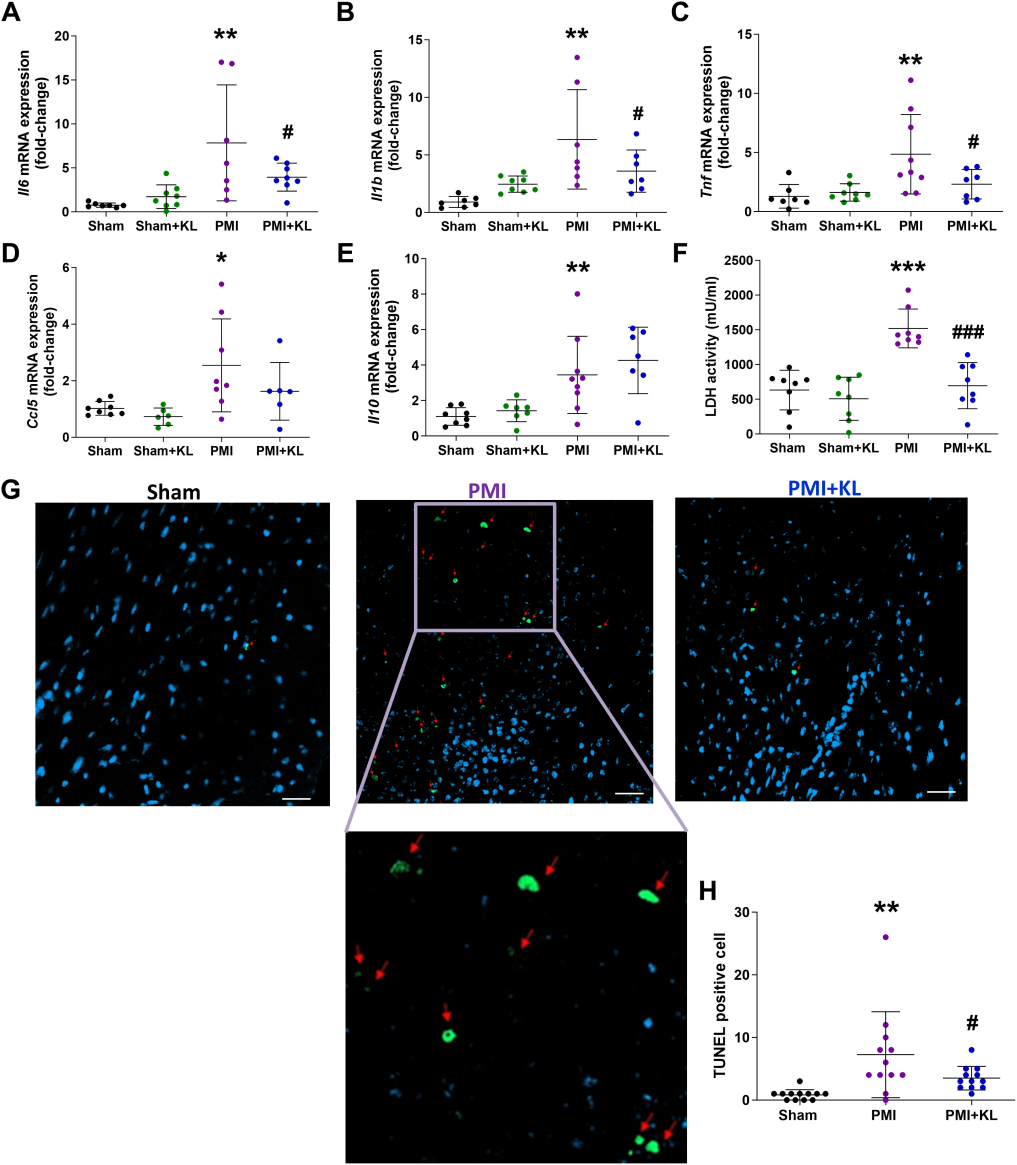
Klotho treatment attenuates cardiac inflammation, necrosis, and apoptosis after MI. (A–E) Mean values of cardiac mRNA expression of interleukins (IL) (A) IL‐6, (B) IL‐1β, (C) tumour necrosis factor (TNF), (D) chemokine (C‐C motif) ligand 5 (CCL5), (E) IL‐10. (F) Plasma lactate dehydrogenase (LDH) activity. (G) Myocardial apoptosis assessed by TUNEL at ×25 magnification, scale bar = 20 μm from left ventricle tissue blocks where TUNEL positive cells were stained in green as indicated by red arrow. (H) TUNEL‐positive cell quantification. *n* = 7–8 sham, *n* = 6–8 sham + KL, *n* = 7–11 PMI, *n* = 6–8 PMI + KL. Histograms represent mean ± SD. *p < 0.05, **p < 0.01, ***p < 0.001 versus sham. #*p* < 0.05, ##*p* < 0.01, ###*p* < 0.001 versus PMI.

Results showed that the pro‐inflammatory cytokines interleukin 6 (*Il6*), interleukin 1 beta (*Il1b*), and tumour necrosis factor (*Tnf*) were significantly elevated in PMI compared to sham mice (Figure 4A–C). Similarly, the chemokine (C–C motif) ligand 5 (*Ccl5*), as a chemokine that activates and directs the movement of certain immune cells such as T cells and macrophages to the heart, was significantly higher in PMI compared to sham mice (Figure 4D). The expression of *Il6*, *Il1b*, and *Tnf* were downregulated after Klotho treatment in PMI mice (Figure 4A–D). Contrastingly, the anti‐inflammatory cytokine *Il10* that was significantly higher in PMI remained elevated in the presence of Klotho treatment (Figure 4E).

Next, we examined necrosis and apoptosis in all experimental groups. LDH is an important oxidoreductase in the anaerobic metabolic pathway and an indicator of acute myocardial necrosis. Thus, systemic LDH activity was significantly higher in PMI than in sham mice but was re‐established to basal levels in the presence of Klotho treatment after MI induction (Figure 4F). Similarly, PMI mice showed a larger number of TUNEL‐staining positive cells compared to sham mice. However, a significant decrease in TUNEL‐stained positive cells was observed in PMI mice after Klotho treatment (Figure 4G,H). All of these data support that recombinant Klotho suppresses inflammatory and maintains the anti‐inflammatory responses reducing the myocardial necrosis and apoptosis in post‐myocardial ischaemic remodelling.

### Recombinant Klotho treatment reduces arrhythmic events after MI both *in vivo* and *in vitro*

**Figure 5:**
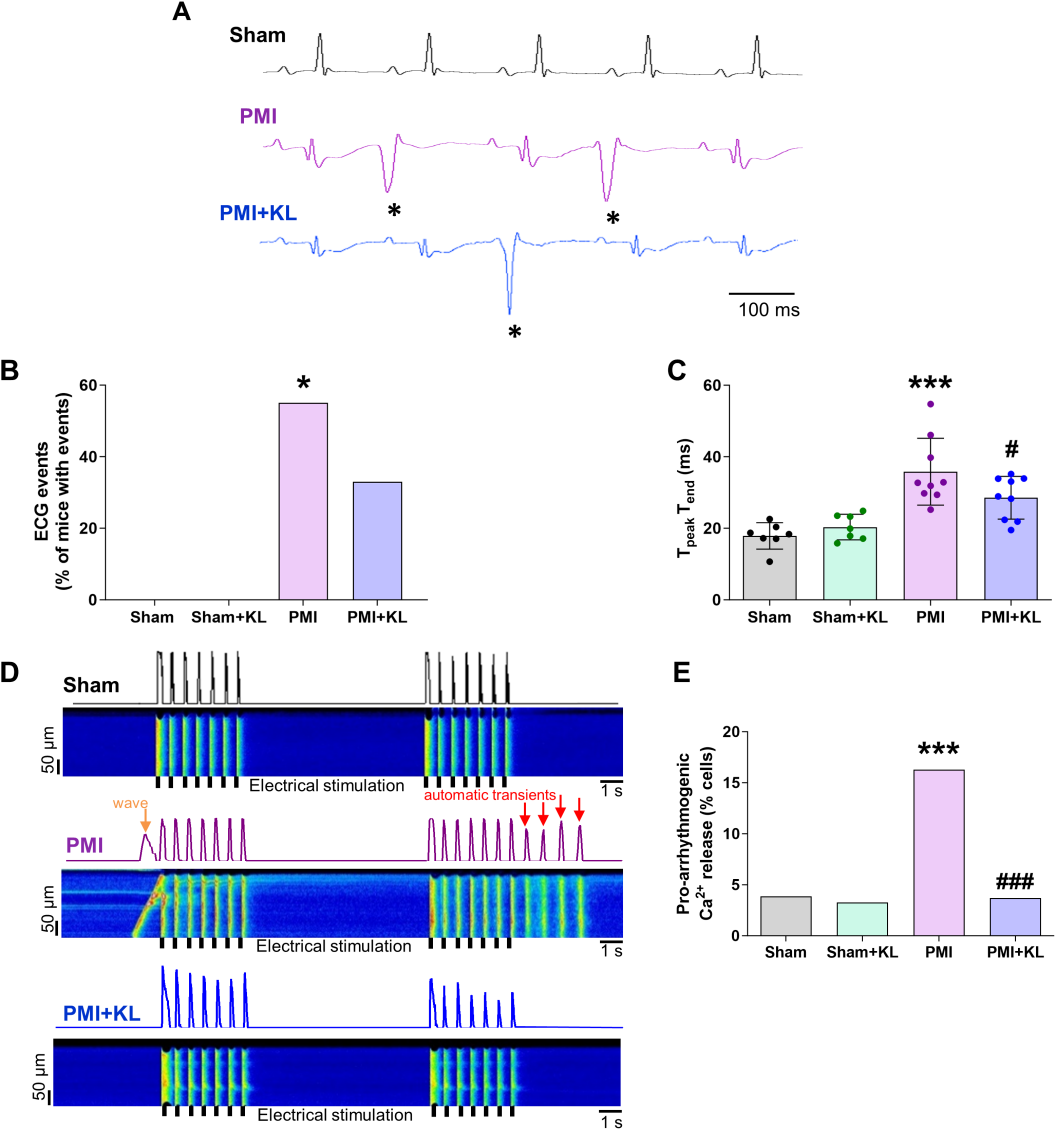
Klotho treatment reduces arrhythmic events in ischaemic cardiomyopathy after MI, under both *in vivo* and *in vitro* approaches. (A) Representative ECG recordings in sham (upper panel), PMI mice (middle panel), and PMI mice treated with Klotho (bottom panel) showing representative ECG events and (B) its occurrence. (C) Mean values of T_peak_T_end_ interval duration (ms); *n* = 7–9 mice per group. (D) Fluorescence profiles (upper panels) and representative line‐scan confocal images (bottom panels) of a cardiomyocyte paced for two cycles from sham, PMI, and PMI + KL (upper to bottom) mice. Black marks indicate electrical stimulation, red arrows automatic transients, and orange arrows automatic Ca^2+^ waves. (E) Percentage of cells with pro‐arrhythmogenic Ca^2+^ events; sham *n* = 4 mice/*n* = 51–60 cells; sham + KL *n* = 5 mice/*n* = 52–61 cells; PMI *n* = 6 mice/*n* = 85–91 cells; PMI + KL *n* = 6 mice/*n* = 75–80 cells. Histograms represent percentage of events in panels C and E. *p < 0.05, ***p < 0.001 versus sham. ^#^ *p* < 0.05, ^###^ *p* < 0.001 versus PMI.

We assessed potential arrhythmic events both *in vivo* by ECG and *in vitro* in cardiomyocytes developed after MI, as well as the effects of Klotho in this setting. Representative 500‐ms lead‐I ECG traces in black correspond to normal ECG recordings from a sham mouse and traces in purple correspond to a PMI mouse, with two examples of premature ventricular contractions (*) and traces in blue corresponding to a PMI + KL mouse and one example of premature ventricular contractions (*) (Figure 5A). Cardiac ECG events occurred more often in PMI mice (55%) than in sham (0%) or sham + KL (0%) mice and decreased in PMI + KL mice (33%) (Figure 5B). Cardiac fibrosis and hypertrophy are strongly linked to arrhythmic disorders, especially in HF developed after MI. We thus analysed the T_peak_T_end_ interval, as changes in its duration are associated with a high likelihood of sudden cardiac death due to fatal ventricular arrhythmias [22, 23]. The T_peak_T_end_ interval was significantly longer in PMI mice than in sham mice (Figure 5C) and was significantly shorter in PMI + KL mice than in PMI mice (Figure 5C). We noted similar arrhythmic effects in isolated cardiomyocytes from each experimental group. We next evaluated pro‐arrhythmogenic events during a specific protocol involving several cycles of seven electrical pulses followed by a recovery period. The number of abnormal Ca^2+^ releases, in the form of spontaneous Ca^2+^ waves or automatic Ca^2+^ transients with spontaneous contractions, and the occurrence of missing Ca^2+^ transients were analysed. Representative line‐scan Ca^2+^ images and cardiomyocyte profiles from each experimental group are shown in Figure 5D, with electrical stimulation (black lines) and the pro‐arrhythmogenic events (red or orange arrows) labelled. The results showed that pro‐arrhythmogenic events were significantly higher in cardiomyocytes from PMI mice (16.3%) than from sham (3.9%) and sham + KL (3.3%) mice. Notably, pro‐arrhythmogenic events were significantly lower (3.7%) in cardiomyocytes from PMI + KL mice than in those from PMI mice (Figure 5E). These results highlight a significant role for Klotho as an anti‐arrhythmic therapeutic strategy after MI.

### Treatment with recombinant Klotho prevents excessive diastolic Ca^2+^ leak in cardiomyocytes after MI through CaMKII and not PKA‐dependent pathways reducing RyR_2_ phosphorylation

**Figure 6:**
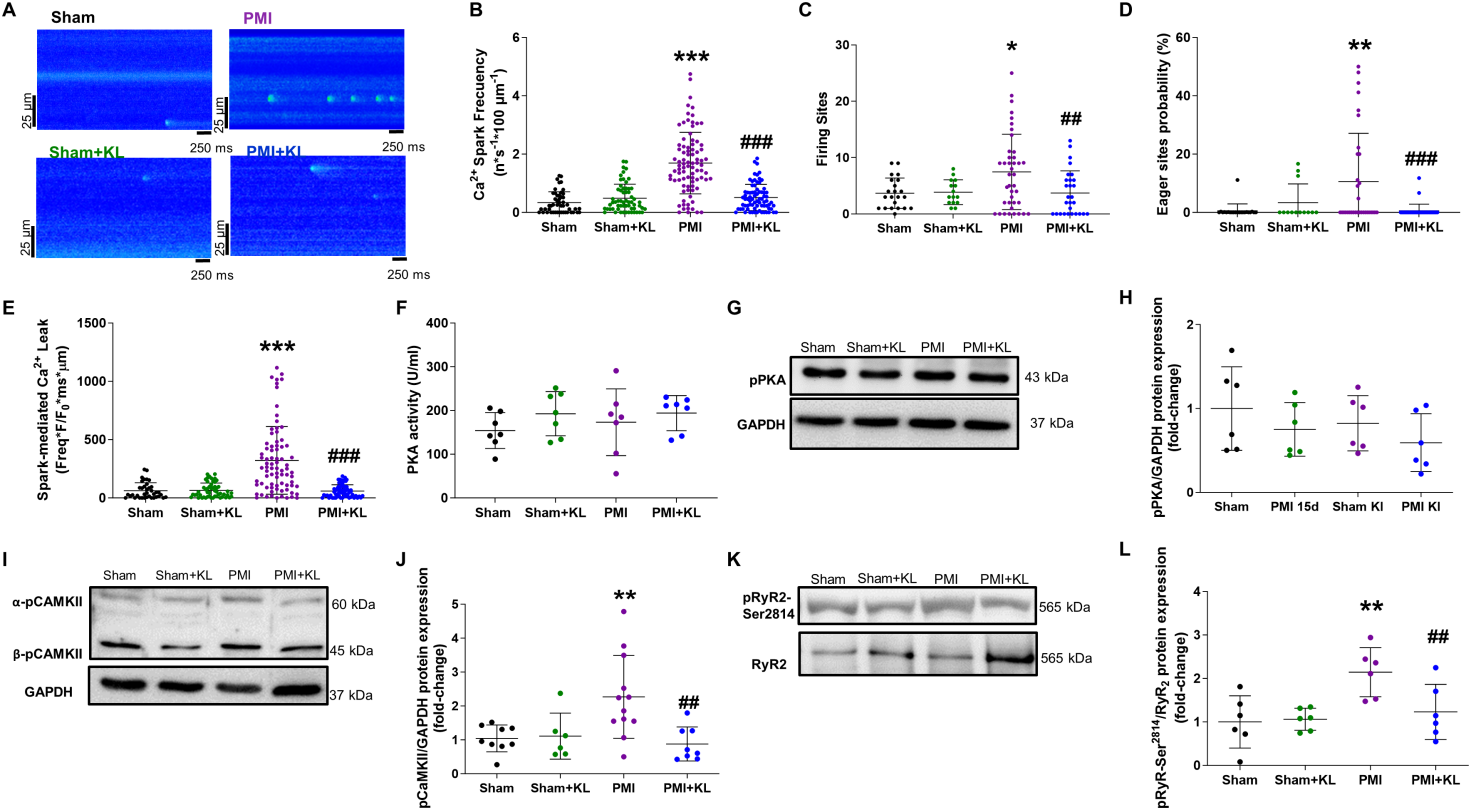
Klotho treatment prevents excessive diastolic Ca^2+^ leak in cardiomyocytes blocking myocardial activation of CaMKII pathway in ischaemic cardiomyopathy after MI. (A) Representative line‐scan confocal images of Ca^2+^ spark recordings obtained in isolated quiescent cardiomyocytes isolated from sham (upper left panel), PMI (upper right panel), sham + KL (bottom left panel), and PMI + KL (bottom right panel) mice. (B) Average data of Ca^2+^ spark frequency (*n ×* s^−1^ *×* 100 μm^−1^), (C) firing sites, and (D) percentage of eager sites of RyR_2_ clusters. (E) Spark‐mediated diastolic Ca^2+^ leak (spark frequency *×* peak *×* duration *×* width). Sham *n* = 4 mice/*n* = 22–49 cells; sham + KL *n* = 5 mice/*n* = 13–60 cells; PMI *n* = 6 mice/*n* = 33–86 cells; PMI + KL *n* = 6 mice/*n* = 34–76 cells. (F) Mean values of PKA activity and (G) representative immunoblots and their quantification (H) of phosphorylated PKA (p‐PKA) in hearts from all experimental groups. (I) Representative immunoblots of phosphorylated CaMKII (p‐CaMKII) and their quantification (J) in hearts from all experimental groups. (K) Representative immunoblots of phosphorylated RyR_2_ (p‐RyR_2_) at Ser^2814^ site and total RyR_2_ (upper panel) and their quantification (L) in hearts from all experimental groups. *n* = 5–9 sham, sham + KL *n* = 5–7, PMI *n* = 4–12, and PMI + KL *n* = 6–8 mice. Histograms represent mean ± SD. *p < 0.05, **p < 0.01, ***p < 0.001 versus sham. ^##^ *p* < 0.01, ^###^ *p* < 0.001 versus PMI.

As high pro‐arrhythmogenic activity in adult cardiomyocytes is frequently associated with increased diastolic Ca^2+^ release, we analysed the release of RyR_2_‐Ca^2+^ under resting conditions in the absence of electrical stimulation, referred to as diastolic Ca^2+^ sparks. Representative line‐scan recordings of Ca^2+^ sparks in quiescent cardiomyocytes from all groups are shown in Figure 6A. Quantification revealed that the frequency of diastolic Ca^2+^ sparks in cardiomyocytes was significantly higher in PMI mice than in sham or sham + KL mice. As with arrhythmogenic events, Klotho treatment significantly prevented the abnormal frequency of diastolic Ca^2+^ sparks in PMI mice (Figure 6B). The increased diastolic Ca^2+^ frequency may be attributed to a greater number of clusters firing Ca^2+^ sparks, to a greater propensity of clusters to fire repeatedly, or to both. We therefore analysed specific sites presenting multiple Ca^2+^ sparks during recording time in each cell. Firing sites were categorised as those where at least one Ca^2+^ spark was recorded. Cardiomyocytes from PMI mice were found to have significantly more firing sites and more active RyR_2_ clusters, termed ‘eager’ clusters, than cardiomyocytes from sham mice (Figure 6C,D). Additionally, PMI mice showed a greater diastolic Ca^2+^ leak (Figure 6E) as an overall measure of spark‐mediated Ca^2+^ release, a value that integrates Ca^2+^ spark frequency with the biophysical properties of each Ca^2+^ spark, including the fluorescence (F/F0), duration (ms), and width (μm) (supplementary material, Table S2). The duration of Ca^2+^ sparks in cardiomyocytes was longer in PMI mice than in PMI + KL mice (supplementary material, Table S2), supporting the idea that Klotho treatment reduces the open state of RyR_2_, which is pathologically enhanced in PMI mice. Thus, overall Ca^2+^ leakage in cardiomyocytes was significantly lower in PMI + KL mice than in PMI mice (Figure 6E). Of note, Klotho treatment normalised these diastolic events (Figure 6B–E). As Klotho treatment prevented Ca^2+^ mishandling in cardiomyocytes, we analysed RyR_2_ phosphorylation, which is one of the main responsible mechanisms linked to ventricular dysfunction and arrhythmia. Several kinases can phosphorylate RyR_2_ and are activated in the setting of HF. We first examined PKA but failed to find significant differences in its enzymatic activity between experimental groups (Figure 6F). We confirmed this by analysing phosphorylated PKA protein levels (Figure 6G,H), finding no significant differences between experimental groups. We next examined Ca^2+^/calmodulin‐dependent kinase type II (CaMKII), a key kinase involved not only in HF but also in ventricular arrhythmias. Our results showed that CaMKII was significantly phosphorylated after 15 days of MI compared to sham mice (Figure 6I,J), which is a clear indication of increased activation. Notably, this was not observed in PMI + KL mice, which had significantly lower levels of CaMKII phosphorylation than PMI mice (Figure 6I,J). Supporting these data, we found a significant increase in RyR_2_ phosphorylation at Ser^2814^, the CaMKII‐specific site, only in PMI mice and not in PMI + KL mice, which showed similar values to those observed in sham mice (Figure 6K,L). These results indicate that the anti‐arrhythmic properties of Klotho are related to the prevention of CaMKII pathway activation.

## Discussion

IHD is the leading cause of cardiovascular mortality worldwide and places a significant burden on healthcare systems [1]. Despite significant progress made in recent decades, there is still a need for a broader understanding of the cellular and molecular mechanisms underlying the pathogenesis of IHD after MI, which could help to improve clinical stratification of patients and provide more effective therapies. We showed that circulating Klotho levels decreased significantly immediately after MI, particularly in STEMI patients with severe ventricular damage. We further showed that exogenous Klotho supplementation had a global cardioprotective effect after MI in a murine experimental model. Overall, our study contributes to a better molecular understanding of the cardioprotective mechanism of Klotho through the specific blockade of the CaMKII pathway (see supplementary material, Figure S3 for a schematic of our study).

Klotho is an anti‐ageing protein with multiple beneficial effects, making it an attractive novel therapeutic strategy. However, despite the considerable interest in Klotho since its discovery over two decades ago, no Klotho‐based therapies have reached clinical trials. We show that the systemic levels of Klotho are profoundly decreased in both STEMI patients and in mice following LAD coronary artery ligation. Klotho is known to decrease with age; however, this did not influence our results, as Klotho levels in STEMI patients were independent of age. Moreover, regression analysis also corroborated that the decrease in circulating Klotho levels observed in STEMI patients were independent of other cardiovascular comorbidities as the presence of DM, hyperlipidaemia, hypertension, or previous IHD. Our results are consistent with previous studies showing that systemic Klotho levels were inversely correlated with congestive HF and MI [22]. However, in the aforementioned study, the observed minimum level of systemic Klotho was ~500 pg/ml, which is significantly higher than that observed in patients in the present study corresponding to the first tertile, 160–476 pg/ml and corresponding to those STEMI patients with the highest mean NT‐proBNP levels (1,587 pg/ml).

In addition, we demonstrated that a marked decrease in systemic Klotho levels in mice was associated with a significant increase in NT‐proBNP levels 15 days after MI, indicating severe HF. However, Klotho exogenous supplementation immediately after MI induction and during the following 15 days prevented this increase in NT‐proBNP levels. However, Klotho levels were not associated with other clinical parameters of cardiac function. The reason of why the decrease in Klotho levels were not associated to clinical parameters of cardiac function may be that this anti‐ageing factor could not be considered a good biomarker of cardiovascular risk whether advanced age is not present, and our cohort of study were adults but not older individuals in which a more drastic decrease in circulating Klotho levels would be expected. These results suggest that increased ventricular damage in IHD is linked to lower systemic Klotho levels, pointing to its usefulness as a biomarker of ventricular damage, and at the same time a potential therapeutic strategy after MI to prevent the deleterious effects of cardiac ischemia.

Having established the relationship between circulating Klotho and NT‐proBNP levels, we investigated the effect of exogenous Klotho on cardiac function using *in vivo* and *in vitro* approaches. CMRI analysis of PMI mice revealed a defect in systolic emptying and heart‐wall thickening, resulting in a lower EF with smaller SV and CO and manifesting a clear cardiac dysfunction. The main consequences of the remodelling mechanisms included infarct expansion, LV dilation, and myocardial thickening, all of which contribute to HF [23, 24]. Klotho treatment prevented these functional changes by significantly reducing the infarct size in PMI mice evidenced by LGE‐CMRI. Gadolinium‐based contrast agents are used to identify areas of irreversible damage, as they are much slower to clear from necrotic and fibrotic tissue than from healthy tissue [23]. These results are supported by other studies [25, 26, 27], reinforcing the idea that Klotho can prevent dysfunctional healing. In addition, recombinant Klotho treatment countered inflammation and cell death induced by permanent cardiac ischemia, supporting Klotho supplementation as a promising therapeutic strategy to globally prevent the deleterious consequences of IHD after a MI.

We next extended these findings by comprehensively analysing the effect of Klotho on cellular function. We found that Klotho treatment prevented the cardiomyocyte hypertrophy evident in PMI mice, likely due to less necrotic tissue in PMI + KL mice, requiring less compensation of the infarcted area. In support of this, the intracellular Ca^2+^ mishandling of cardiomyocytes from PMI mice was indicative of a compensated hypertrophy phenotype, with a significant increase in systolic Ca^2+^ release and cellular contraction, as the unaffected ventricular tissue must functionally compensate for the remaining dead ventricular tissue. This was not necessary in Klotho‐treated cardiomyocytes from PMI mice, probably because of the smaller infarct area. Thus, the increase in systolic Ca^2+^ transients and cardiomyocyte shortening was not observed in Klotho‐treated PMI mice. To date, the cardioprotective effect of Klotho on cardiomyocytes has only been observed in conditions of uremic cardiomyopathy [18, 19, 20], as its expression is significantly reduced in renal disease. Here, we demonstrate for the first time the beneficial effects of Klotho on cardiomyocyte function in conditions of preserved renal function, as PMI mice maintained normal renal function (data not shown). In addition, the reduction in Klotho levels observed in STEMI patients was not associated with renal dysfunction, as both cohorts had an estimated glomerular filtration rate of ≥90 ml/min/1.72 m^2^.

We observed that Klotho treatment prevented electrical changes in PMI mice, including prolongation of the QRS interval. QRS complex duration is associated with intraventricular conduction and its increase might indicate slow action potential propagation due to increased LV mass and fibrosis [28]. In addition, prolongation of QT and JT intervals indicates altered cardiac repolarisation, which could trigger ventricular arrhythmias [28]. Klotho treatment prevented these alterations in cardiac depolarisation and repolarisation, supporting its potential as an anti‐arrhythmic agent after MI. Previous studies demonstrated antifibrotic and anti‐inflammatory effects of Klotho due to its nature as an inducer of cellular autophagy [26], which cannot be discounted in the present study. Moreover, we showed that Klotho treatment prevented prolongation of the T_peak_T_end_ interval and reduced arrhythmic events observed after MI. Prolongation of the T_peak_T_end_ interval is linked to a higher likelihood of fatal ventricular arrhythmias and sudden cardiac death [29, 30]. This is clinically relevant because ventricular arrhythmias often trigger sudden cardiac death in the acute convalescent phase after MI [31]. In support of this, we observed a reduction in mortality in PMI mice treated with Klotho (13% reduction, data not shown) compared with untreated PMI mice.

Beyond the antifibrotic and anti‐hypertrophic actions of Klotho, it is important to note that Klotho exerted beneficial effects directly on cardiomyocytes. Indeed, compared with untreated PMI mice, Klotho‐treated PMI mice had fewer arrhythmic events not only *in vivo*, but also at the cardiomyocyte level. One of the major cellular mechanisms involved in HF is increased diastolic Ca^2+^ release, as evidenced by RyR_2_ hyperphosphorylation and SR Ca^2+^ leak, which are directly linked to CaMKII [32, 33] or PKA [34] activation. We found that PKA was not activated after MI, at least in the time frame of our study. These results are consistent with previous studies, which showed that PKA‐mediated RyR_2_ phosphorylation had little or no functional relevance for RyR_2_‐mediated Ca^2+^ leak when SR Ca^2+^ levels remained constant [35], as observed in our study. However, we observed increased CaMKII phosphorylation and RyR_2_ hyperphosphorylation at the CaMKII site after MI. RyR_2_ hyperactivity is described as a high frequency of diastolic Ca^2+^ sparks and a propensity of some RyR_2_ clusters to fire Ca^2+^ repeatedly (eager clusters) and to activate neighbouring RyR_2_ clusters throughout the entire cardiomyocyte. All of these physiological and molecular events associated with CaMKII activation and RyR_2_ hyperphosphorylation at the CaMKII site were prevented by Klotho treatment. The anti‐arrhythmogenic effect of Klotho related to diastolic Ca^2+^ leak has also been described in several models of uremic cardiomyopathy, where renal function was severely impaired as a consequence of the development of chronic kidney disease or the induction of acute kidney injury [18, 20]. Our study describes for the first time the anti‐arrhythmic cellular effects of Klotho treatment, dependent on intracellular RyR_2_ modulation, beyond the renal setting and in the context of IHD. These anti‐arrhythmic together with antihypertrophic and antifibrotic Klotho actions suggest that it may operate in a global upstream level. Supporting this idea, we discovered that Klotho treatment was able to prevent the upregulated β‐adrenergic signalling observed after MI (data not shown). Future studies will be necessary to corroborate the anti‐β‐adrenergic actions of Klotho in the heart. Clinical advances in the management of MI have successfully reduced post‐MI mortality, although this is associated with an increased morbidity as a consequence of the development of ischaemic HF. Consequently, there is growing interest in developing more effective strategies focused on preventing post‐MI complications. Redirecting efforts towards reducing HF burden, thereby enhancing overall quality of life and increasing disease‐free years, may provide a major benefit to patients who have suffered a MI.

However, this study has some limitations such as the absence of baseline levels of Klotho in STEMI patients due to these patients arrived in an unplanned form at the emergency service of our hospital and without any previous clinical registry. However, experimental data in PMI mice support the idea that a reduction in circulating Klotho levels depends exclusively on myocardial ischaemia as no other cardiovascular or renal comorbidities were present. Future studies will be required to determine the mechanisms by which systemic Klotho levels decline after a cardiac ischaemic event. Our study underscores the potential of exogenous Klotho supplementation as a novel protective measure against ischaemic HF, reducing the development of detrimental cardiac remodelling and of critical post‐MI complications, such as ventricular arrhythmia. Recombinant Klotho treatment prevented CaMKII‐dependent pathway activation and RyR_2_ hyperphosphorylation, thereby ameliorating the intracardiomyocyte Ca^2+^ mishandling and diastolic SR Ca^2+^ leak observed in IHD. Our translational findings support the potential importance of Klotho as a new biomarker of post‐MI ventricular injury, even in the setting of preserved renal function and also as a novel therapeutic strategy for the prevention of cardiac events associated with IHD. Further specific studies in humans are needed to more definitively assess the relevance of Klotho treatment in the clinical setting of MI and its consequences.

## Author contributions statement

GR‐H and SV‐S designed the study and experiments. SV‐S, AB, PF‐C, AM, SP and ER‐S performed human study and analysis of clinical data. SV‐S and PC performed histological analysis. SV‐S and EM‐G performed experimental MI model in mice. SV‐S, LG‐L, JP, DG‐M and EM‐G performed intracellular calcium recordings in isolated cardiomyocytes. SV‐S, IG‐C and EM‐G performed biochemical analysis and western blotting. MF‐V and GR‐H had direct access and verified data from experimental MI models in mice. GR‐H, SV‐S, AB and PC had direct access to clinical data and verified all the analyses. GR‐H, MF‐V, MV and LMR interpreted the results. GR‐H supports all the funding acquisition, project administration and supervision. SV‐S and GR‐H wrote the manuscript. All authors reviewed and edited the manuscript.

## Supporting information


Supplementary materials and methods
**Figure S1.** Circulating Klotho levels in ST‐segment elevation myocardial infarction (STEMI) patients in presence or absence of either (A) DM, (B) hyperlipidaemia, (C) hypertension, or (D) previous IHD
**Figure S2.** Klotho treatment prevents changes in intra‐cardiomyocyte Ca^2+^ removal function in ischaemic cardiomyopathy after MI
**Figure S3.** Schematic diagram of cardioprotective mechanism of Klotho through specific blockade of CaMKII pathway in our study
**Table S1.** Linear regression analysis of plasma KL levels and comorbidities
**Table S2.** Ca^2+^ spark characteristics in cardiomyocytes from Sham, PMI, Sham + KL, and PMI + KL mice

## Data Availability

The data and materials that support the findings of this study are available from the corresponding author upon reasonable request.
