# Supplementary material for: Recombinant Klotho administration after myocardial infarction reduces ischaemic injury and arrhythmias by blocking intracellular calcium mishandling and CaMKII activation
Source: J Pathol. 2025 Jan 15;265(3):342–56. doi: 10.1002/path.6388 (PMC11794962; doi:10.1002/path.6388)
Supplement: Supplementary file 1 — Supplementary materials and methods Figure S1. Circulating Klotho levels in ST‐segment elevation myocardial infarction (STEMI) patients in presence or absence of either (A) DM, (B) hyperlipidaemia, (C) hypertension, or (D) previous IHD Figure S2. Klotho treatment prevents changes in intra‐cardiomyocyte Ca2+ removal function in ischaemic cardiomyopathy after MI Figure S3. Schematic diagram of cardioprotective mechanism of Klotho through specific blockade of CaMKII pathway in our study Table S1. Linear regression analysis of plasma KL levels and comorbidities Table S2. Ca2+ spark characteristics in cardiomyocytes from Sham, PMI, Sham + KL, and PMI + KL mice [file PATH-265-342-s001.docx]

**Recombinant Klotho administration after myocardial infarction reduces ischaemic injury and arrhythmias by blocking intracellular calcium mishandling and CaMKII activation**

S Vázquez-Sánchez *et al. J Pathol* <https://doi.org/10.1002/path.6388>

**Supplementary materials and methods**

**Supplementary Figures S1–S3**

**Supplementary Tables S1-S2**

**Supplementary materials and methods**

**Post-myocardial infarction (PMI) experimental model**

Adult C57BL/6J mice purchased from Charles River Laboratories International (Wilmington, MA, USA) were maintained in temperature- and humidity-controlled rooms on a 12-h light/dark cycle with *ad libitum* access to water and a standard diet (ROD14, Altromin Spezialfutter GmbH & Co., Lage, Germany). Animals were housed in groups of four per cage of 553 cm^2^ × 20.8 cm depth (polysulfone cage type II L, SODISPAN) with standard wood shavings (ECO-PURE 7 hips, Tapvei®). PMI was performed surgically under isoflurane anaesthesia (induction at 3% and maintenance at 1.5% v/v, isoflurane/oxygen). For pre-operative analgesia, meloxicam (Metacam®, 2 mg/kg) and buprenorphine (Buprex®, 0.1 mg/kg) were applied subcutaneously. For animal preparation, depilatory cream was applied on the throat and the left side of the thorax and ointment to the animals' eyes. Excess fur was removed and cream was applied with gauze, and the exposed skin was cleaned with povidone-iodine. The unconscious mouse was placed in the supine position on a heating pad. A lack of response was confirmed by toe pinch, and limbs were secured with adhesive tape. The mouse was intubated by tracheostomy and connected to a small animal ventilator (Inspira Advanced Safety Ventilator, Pressure Controlled) for artificial ventilation. The ventilator was set at mouse weight (kg) in pressure-controlled mode with 2 ml PEEP. The mouse was placed under a microsurgery stereo microscope, and a 1-cm incision was made parallel to the lower costal edge. Curved forceps were used to gently separate the fascia of the pectoralis muscles until the ribs were exposed. Left thoracotomy was performed in the third intercostal space to visualise the anterior surface of the heart and left lung. The ribs were separated with 7/0 blue monofilament polypropylene non-absorbable suture. The pericardium was carefully removed without harming the heart and lungs. The left anterior descending (LAD) coronary artery was located as a superficial bright red line running from the edge of the left auricle towards the apex of the heart. The left coronary artery was ligated with 8/0 black monofilament polyamide non-absorbable suture. The lower left side of the left ventricle instantly turned pale upon ligation. The chest after filling with saline solution and skin were closed with 7/0 blue monofilament polypropylene non-absorbable suture. Mice were kept on a heating pad with 100% oxygen administration until recovery.

**Cardiac magnetic resonance imaging (CMRI)**

The instrument was equipped with a gradient coil that provides a gradient strength of 450 mT/m. Data were acquired using the software package Paravision 6.0.1pl3 ICON (Bruker BioSpin GmbH). Mice were anaesthetised with a mixture of isoflurane and oxygen. Heart rate (HR) and respiration were monitored and used to trigger image acquisition with a 1025 SAM monitoring and gating system (SA Instruments, New York, NY, USA). Several gradient echo images with different orientations were acquired to localise the short-axis planes. Once the short axis was set, cardiac and respiratory-triggered FLASH sequence was used for quantification of myocardial infarction (MI) and functional/volumetric measurements. A total of seven experiments of one slice with variable location were acquired to cover the complete heart (from the bottom of the apex until the end of the ventricles). This dataset was acquired with a field of view = 2.25 × 2.25 cm^2^, slice thickness = 1.25 mm, and spatial resolution = 150 × 150 µm^2^. We used late gadolinium enhancement (LGE) to obtain the maximal contrast between the infarcted and viable myocardium with an i.p. injection of 56 mg/kg gadoteridol (ProHance, Bracco International, Amsterdam, the Netherlands). For MI evaluation, the LGE experiments were acquired with a repetition time (TR) = 100 ms; an echo time (TE) = 2.2 ms; a flip angle (α) = 75° and number of averaged images (NA) = 8. For each slice of the cine-cardiac sequence, the left ventricle (LV) was segmented in images corresponding to diastole and systole. The LV volume for each slice was summed to obtain the total LV volume at the end of diastole and systole (EDV and ESV respectively). Ejection fraction (EF), stroke volume (SV), cardiac output (CO), and wall thickening (WT) were obtained from these data applying the following formulae: EF = [LVEDV − LVESV/LVEDV] × 100; SV = EDV − ESV; CO = SV/1000 *×* HR, WT= [WTD − WTS/WTD] × 100. For functional measurements, a white blood CINE sequence was used to evaluate heart function of the whole heart. Ten images per cardiac cycle were acquired to cover the entire cardiac cycle. TR was variable depending on the animal's heart and respiration rate. Other imaging parameters were as follows: TE = 2.2 ms, α = 45°, and NA = 10. The heart rate for each mouse was calculated as the mean value of the heart rate during the entire CINE experiment. CMRI images were analysed with ImageJ version 1.49 (NIH, MD, USA).

**ECGs**

ECGs were recorded from mice lightly anaesthetised with 1.5% v/v, isoflurane/oxygen in prone position on a preheated pad at 37 °C. Registries were obtained within 10 min under basal condition (before surgical procedure) and 15 days after surgical procedure. Files were analysed using LabChart 7.0 software (ADInstruments, Sydney, Australia). QRS, QT, JT, T_peak_T_end_ intervals, and cardiac events were obtained from the LabChart analysis. QT interval duration was corrected using the Mitchell formula:


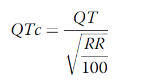
,

where QTc is the corrected QT interval, QT is the interval between Q wave and T wave end, and RR is the interval between one R wave and the R wave of the next QRS complex.

**Adult mouse ventricular cardiomyocyte isolation**

Ventricular cardiomyocytes were isolated from hearts of adult C57BL/6J mice after 15 days of surgical procedure. Mice were sacrificed with sodium pentobarbital–heparin (100 mg/kg 4 UI/g, i.p.), and hearts were quickly removed and cannulated via the ascending aorta on a Langendorff perfusion system. Hearts were retrograde perfused with calcium-free Tyrode’s solution supplemented with 0.2 mM ethylene glycol tetraacetic acid (EGTA) for 3–5 min at 37 ºC, followed by 2–3 min perfusion with Tyrode’s solution supplemented with 0.1 mM CaCl2, 1 mg/ml type II collagenase (Worthington, Lakewood, NJ, USA), and 1 mg/ml bovine serum albumin (BSA). The ventricles were removed and cut into pieces and mechanically dissociated in the enzymatic solution. The resulting ventricular cardiomyocyte cell suspension was filtered through a nylon mesh strainer (250 μm) to remove pieces of tissue and finally centrifuged at room temperature at 300 rpm for 3 min. The supernatant was discarded and the pellet resuspended in Tyrode’s solution containing 0.5 mM CaCl_2_ and 2 mg/ml BSA, and centrifuged again under the same conditions, and finally the resultant pellet was resuspended in a storage Tyrode solution containing 1 mM CaCl_2_ and 2 mg/ml BSA. The Tyrode solution composition was as follows (in nM): 130 NaCl, 5.4 KCl, 0.4 NaH_2_PO_4_, 0.5 MgCl_2_, 35 HEPES, and 22 glucose, and pH was adjusted to 7.4 using LiOH.

**Intracellular Ca^2+^ imaging**

Isolated ventricular rod-shaped cardiomyocytes, excitable under electrical stimulation and tolerant to Ca^2+^, were used. Cardiomyocytes were preloaded with the Ca^2+^-sensitive fluorescent dye Fluo-3AM (5 μM; Invitrogen, Carlsbad, CA, USA) for 30 min at room temperature. Images were obtained with a MetaZeiss LSM 510 confocal microscope using a ×40 water-immersion objective and numerical aperture of 1.2. A line was traced across the longitudinal axis of Fluo-3AM-loaded cells placed in a field stimulation chamber with two parallel platinum electrodes and filled with Tyrode's solution. Local increases in cytosolic intracellular Ca^2+^ transients were analysed in cardiomyocytes electrically stimulated at 2 Hz. Fluorescence (F) values were normalised to the basal fluorescence (F_0_) to obtain the fluorescence ratio (F/F_0_). The decay time constant of Ca^2+^ transients (*Tau*) was obtained by fitting the decay time trace to a single exponential and corresponded to the kinetics of the relaxation phase, which was mainly due to the re-uptake of Ca^2+^ into the sarcoplasmic reticulum (SR) by the SERCA_2a_ pump. Cell shortening profiles were obtained offline by measuring cell length at rest and during electrical stimulation from each line of Ca^2+^ transient images. The SR Ca^2+^ load was estimated as the amplitude of caffeine-induced Ca^2+^ transients (10 mM caffeine). SR fractional Ca^2+^ release was also analysed to determine the SR Ca^2+^ reserve available for each cardiac contraction and was measured by normalising the steady state of the Ca^2+^ transient (peak F/F_0_) by the caffeine-evoked intracellular Ca^2+^ transient (peak F/F_0_ evoked by rapid caffeine application). Quiescent Fluo-3AM-loaded cells were scanned to record spontaneous Ca^2+^ sparks due to the opening of the ryanodine receptor (RyR_2_) when electrical stimulation was stopped. Spontaneous Ca^2+^ sparks were defined as localised, rapid, and brief elevations in Ca^2+^ fluorescence of at least four times the standard deviation of F_0_ after Ca^2+^ transient recordings. Pro-arrhythmogenic Ca^2+^ events were considered to be any spontaneous abnormal release of Ca^2+^ as waves, missing transients, or automatic contractions during a protocol with several cycles of seven electric pulses followed by a recovery period. Confocal Ca^2+^ images were analysed using home-made routines in Interactive Data Language (IDL; Research Systems, Boulder, CO, USA) and ImageJ version 1.49 (NIH, USA) software programs.

**RNA isolation and RT-qPCR**

Frozen heart tissues from mice were pulverised and total RNA was extracted using the RNeasy Mini Kit (Qiagen., Hilden, Germany). RT-qPCR was performed using the FastStart Essential DNA Green Master (Roche, Basel, Switzerland) on the LightCycler® 480 II instrument (Roche). Primers were designed using PrimerBlast Software (www.ncbi.nlm.nih.gov/tools/primer-blast), and reactions were performed in duplicate. Relative gene expression was evaluated using the 2^−ΔΔCt^ method and normalised using the endogenous gene transcripts Ribosomal Protein Lateral Stalk Subunit P0 (*Rplp0*).

Quality and quantity of RNA were assessed using the NanoDrop One Microvolume UV-Vis Spectrophotometer (Thermo Fisher Scientific., Waltham, MA, USA), and 4 μg of total RNA was reverse-transcribed to cDNA using the High-Capacity cDNA Reverse Transcription Kit (Applied Biosystems, Foster City, CA, USA).

Primer sequences are: *Rplp0*-Forward AGATGCAGCAGATCCAT; *Rplp0-*Reverse GTTCTTGCCCATCAGCACC; collagen type 1 alpha 1 chain (*Col1a1)*-Forward AATGGCACGGCTGTCTGCGA; *Col1a1*-Reverse AGCACTCGCCCTCCCGTCTT; collagen type 3 alpha chain (*Col3a1)*-Forward CTGTAACATGGAAACTGGGGAAA; *Col3a1*-Reverse CCATAGCTGAACTGAAAACCACC; myosin heavy chain 7 (*Myh7)-*Forward TTCTACTCTGCGCTGGCTTT; *Myh7-* Reverse GGCTGAGCCTTGGATTCTCA; myosin heavy chain 6 (*Myh6)-* Forward TAAAGGGGCTGGAGCACTGA; *Myh6-* Reverse CGTCCGTCATTCTGTCACTCA; IL-6 (*Il6)*-Forward AGCCAGAGTCCTTCAGAGAGAT; *Il6*-Reverse TGGAAATTGGGGTAGGAAGGAC; IL-1 beta *(Il1b)*- Forward TGCCACCTTTTGACAGTGATG; *Il1b* -Reverse GTGCTGCTGCGAGATTTGAA; *Tnf*-Forward TAGCCCACGTCGTAGCAAAC; *Tnf*-Reverse ACAAGGTACAACCCATCGGC; C-C motif chemokine ligand 5 (*Ccl-5*)-Forward ACCATATGGCTCGGACACCA; *Ccl-5*-Reverse CTTGGCGGTTCCTTCGAGT; IL-10 (*Il10)*-Forward GCTGTCATCGATTTCTCCCCT; *Il10*-Reverse CATTCATGGCCTTGTAGACACC.

**Western blotting**

Frozen heart tissues from mice were pulverised and heart proteins were extracted in a lysis buffer containing 0.05 M Tris, 0.32 M sucrose, 0.5% CHAPS, 0.5 µM okadaic acid, and protease inhibitors 0.1 M PMSF, 12 μM leupeptin, 0.2 μM aprotinin, and 0.5 M benzamidine. Homogenates were centrifuged at 4,500 rpm for 10 min at 4 ºC. Proteins were separated by SDS-PAGE electrophoresis and transferred to PVDF membranes (Trans-Blott®, 0.2 µm PVDF, Bio-Rad Laboratories, Hercules, CA, USA) on a semi-dry transfer system. Membranes were blocked with 5% BSA for the phosphorylated primary antibodies phospho-RyR-Ser^2814^, phospho-calcium/calmodulin-dependent protein kinase II (CaMKII), and phospho-protein kinase A (PKA) and with 5% milk for the remaining primary antibodies, total RyR_2_, glyceraldehyde-3-phosphate dehydrogenase (GAPDH), and α-Klotho, in 0.1% Tween 20 for 1 h, incubated overnight at 4 °C, and then incubated with an appropriate HRP-conjugated secondary antibody for 1 h. Immunoreactive bands were visualised by chemiluminescence using the ECL substrate (Thermo Fisher Scientific). Bound antibody was visualised using an ImageQuant LAS4000 imaging system (GE Healthcare, Marlborough, MA, USA), and densitometry was performed with ImageQuant TL 8.1. For plasma WB a Ponceau S (A40000279, Thermo Fisher Scientific) staining-based dot-blot assay was used for protein quantification.

Primary antibodies used were as follows: rabbit polyclonal IgG anti-p-CAMKII (A010-50AP Badrilla, Leeds, UK), rabbit polyclonal IgG anti-p-RyR-Ser2814 (A010-31 Badrilla) at 1:1,000 dilution, mouse monoclonal IgG1 anti-RyR (MA3-916 Thermo Fisher Scientific) at 1:1,000 dilution, goat polyclonal IgG anti-α-Klotho (AF1819, R&D Systems, Inc.) at 1:1,000 dilution, mouse monoclonal IgG anti-p-PKA (sc-377575, Santa Cruz Biotechnology, Dallas, TX, USA) at 1:250 dilution, and mouse monoclonal IgG1 anti-GAPDH (AM4300, Thermo Fisher Scientific) at 1:200,000 dilution. HRP-conjugated secondary antibodies used were as follows: anti-mouse IgG kappa at 1:5,000 dilution, mouse anti-goat at 1:5,000 dilution, and mouse anti-rabbit at 1:5,000 dilution (sc-516102 and sc-2357, respectively, both from Santa Cruz Biotechnology).

**PKA activity**

PKA activity was measured with a colorimetric assay (Thermo Fisher Scientific). Twenty micrograms of total protein from homogenised hearts were obtained in a lysis buffer containing 0.05 M Tris, 0.32 M sucrose, 0.5% CHAPS, 0.5 µM okadaic acid, and protease inhibitors (0.1 M PMSF, 12 µM leupeptin, 0.2 µM aprotinin, and 0.5 M benzamidine).

**Supplementary Figures S1–S3**


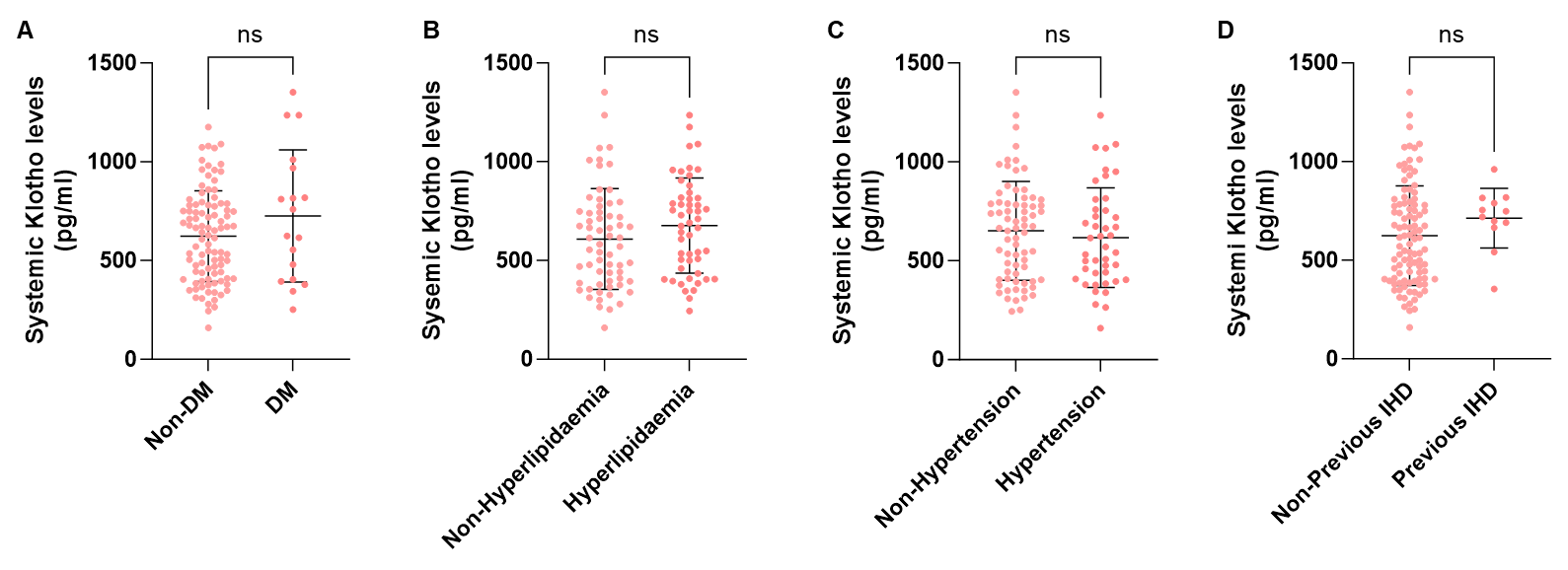


**Figure S1.** Circulating Klotho levels in ST-segment elevation myocardial infarction (STEMI) patients in presence or absence of (A) diabetes mellitus (DM), (B) hyperlipidaemia, (C) hypertension, or (D) previous IHD. ns = not significant.


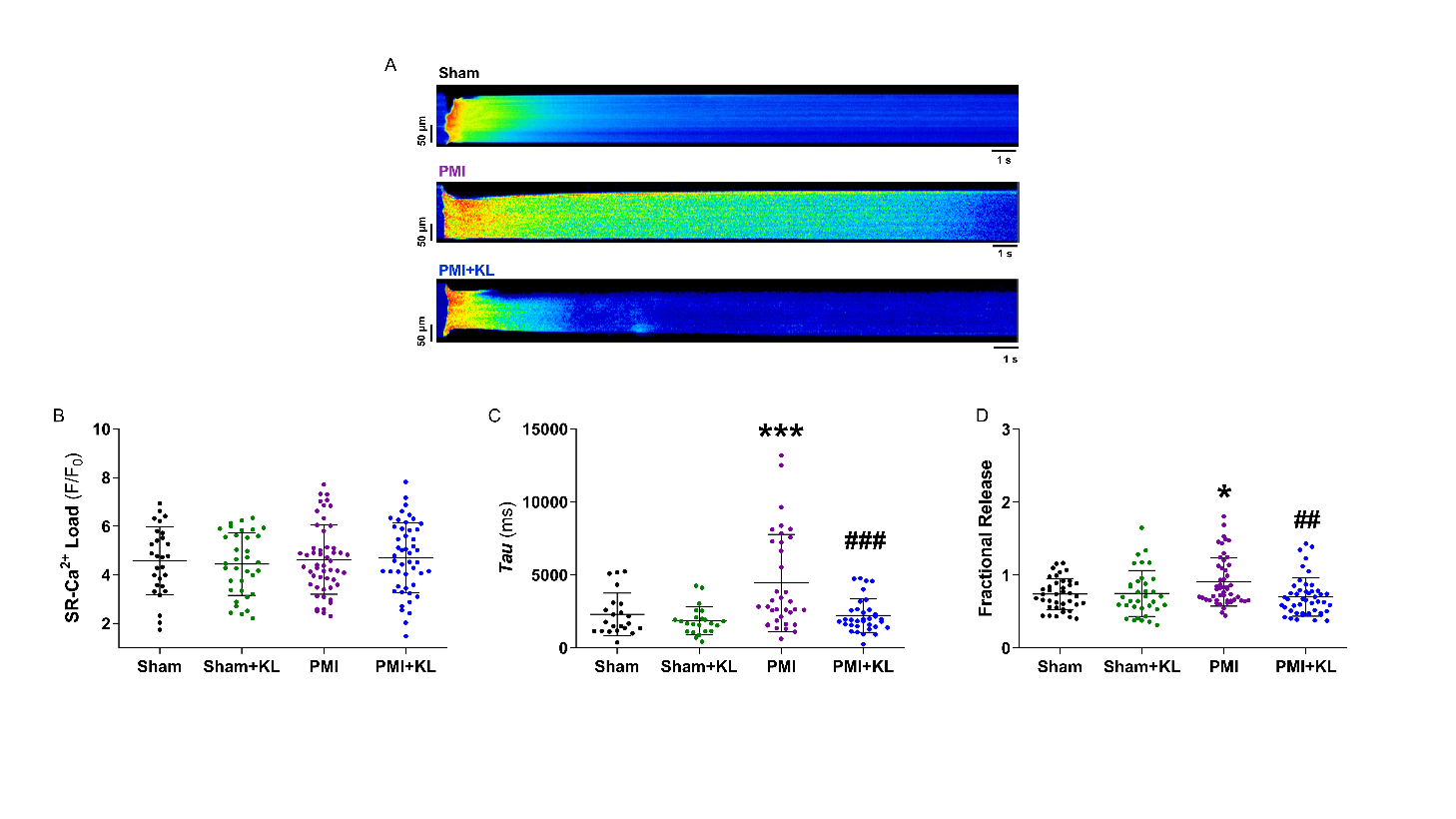


**Figure S2.** Klotho treatment prevents changes in intra-cardiomyocyte Ca^2+^ removal function in ischaemic cardiomyopathy after MI. (A) Representative line-scan images of caffeine-evoked Ca^2+^ transients in cardiomyocytes from sham (upper panel), PMI (middle panel), and PMI + Klotho treatment (bottom panel) mice obtained by confocal microscopy. (B) Mean values of amplitude of caffeine-evoked intracellular Ca^2+^ transients (Ca^2+^ fluorescence peak, F/F_0_). (C) Mean decay time constant of caffeine-evoked Ca^2+^ transient (*Tau*, ms). (D) Fractional Ca^2+^ release from SR (FF_0_ Ca^2+^ transients/FF_0_ Ca^2+^ transient amplitude evoked by caffeine). Histograms represent mean ± SD. Sham *n* = 4 mice/*n* = 22–39 cells, sham+KL *n* = 4 mice/*n* = 23–35 cells, PMI *n* = 6 mice/*n* = 35–52 cells, and PMI+KL *n* = 6 mice/*n* = 34–48 cells. **p* < 0.05, ****p* < 0.001 versus sham. ^##^*p* < 0.01, ^###^*p* < 0.001 versus PMI.

**
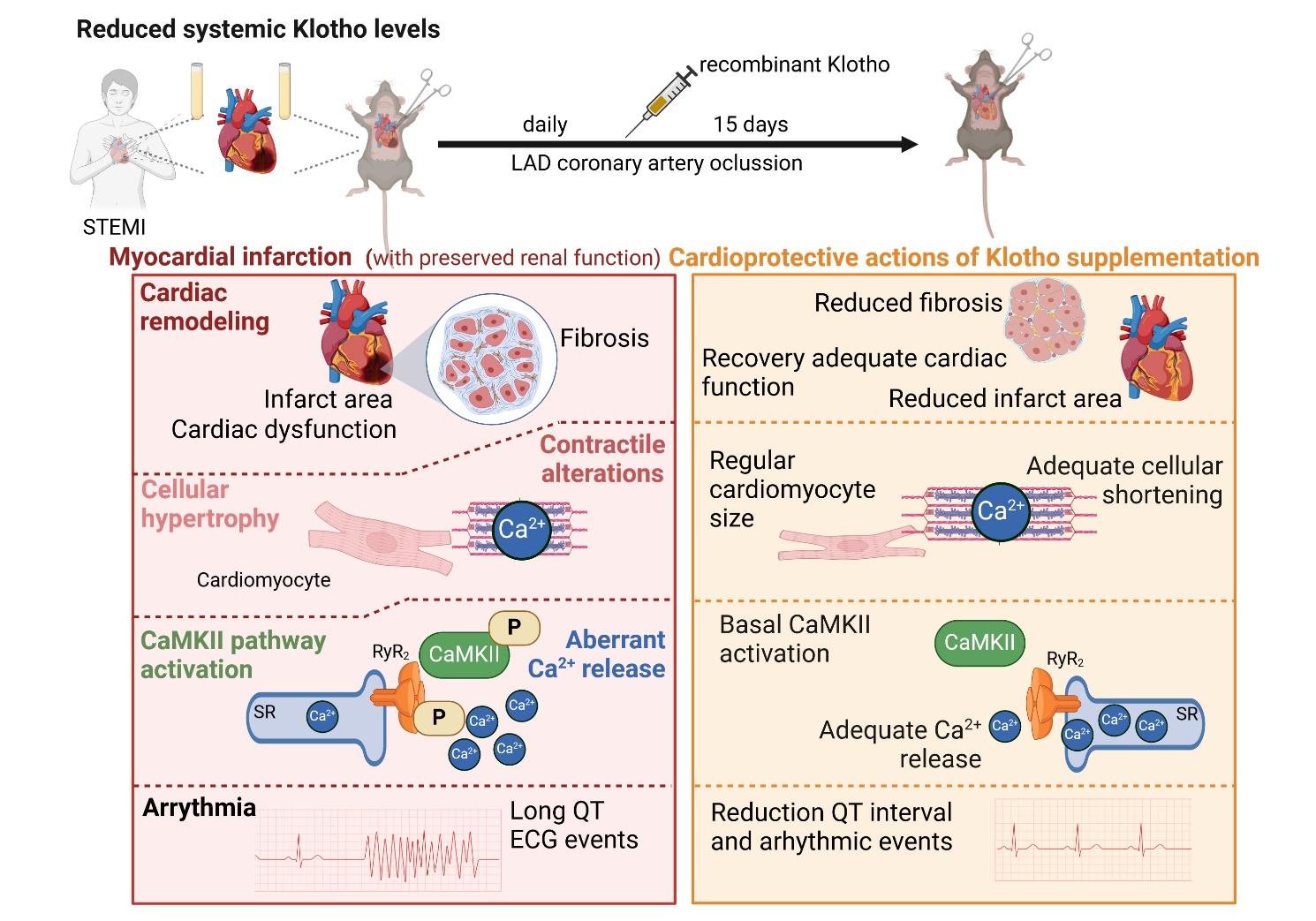
**

**Figure S3.** Schematic diagram of cardioprotective mechanism of Klotho through specific blockade of CaMKII pathway in our study. Klotho is an anti-ageing factor traditionally investigated in kidney. Here we demonstrated that patients with STEMI and a murine model of MI showed decreased circulating Klotho levels. Moreover, decreased circulating Klotho was associated with greater ventricular damage after MI, even in absence of impaired renal function. We hypothesised that Klotho supplementation could contribute to the prevention of IHD after MI, even under conditions of preserved renal function. Here we showed that Klotho treatment in mice with MI prevented cardiac remodelling, pro-arrhythmic events, and intracardiomyocyte calcium (Ca2^+^) mishandling through blocking Ca2^+^/calmodulin kinase type II (CaMKII) pathway activation, supporting its utility as a potential therapy for IHD. In conclusion, Klotho supplementation after MI protects the heart and cardiomyocytes from adverse cardiac remodelling, thereby representing a potential new treatment for IHD. Created with BioRender.com.

**Supplementary Tables S1–S2**

**Table S1.** Linear regression analysis of plasma KL levels and comorbidities

| **Linear regression analysis** | | | |
| --- | --- | --- | --- |
|  | **β** | **95% CI** | ***P*-value** |
| **DM** | 102.29 | −24.13 – 228.72 | 0.112 |
| **Hypertension** | -34.41 | −130.38 – 61.58 | 0.479 |
| **Hyperlipidaemia** | 67.97 | −24.93 – 160.87 | 0.150 |
| **Previous IHD** | 128.94 | −15.74 – 273.63 | 0.108 |

DM, diabetes mellitus; IHD: ischaemic heart disease

**Table S2.** Ca^2+^ spark characteristics in cardiomyocytes from sham, PMI, sham+KL, and PMI+KL mice

| **Parameter** | **Sham** | **PMI** | **Sham+KL** | **PMI+KL** | |
| --- | --- | --- | --- | --- | --- |
| Duration (ms) | 28.96±9.04 | 37.84±11.88*** | 27.47±9.24 | | 31.44±9.99^###^ |
| Width (µm) | 3.35±0.65 | 3.06±0.95 | 2.91±0.83 | | 3.12±0.67 |
| Peak (F/F_0_) | 1.59±0.23 | 1.58±0.19 | 1.47±0.15 | | 1.54±0.21 |

Sham *n* = 4 mice/*n* = 49 cells and 275 sparks, sham+KL *n* = 5 mice/*n* = 60 cells and 318 sparks, PMI *n* = 6 mice/*n* = 86 cells and 1,066 sparks, and PMI+KL *n* = 6 mice/*n* = 76 cells and 476 sparks. ****p* < 0.001 versus sham ^###^*p* < 0.001 versus PMI.
